# Extracellular vesicles carry transcriptional ‘*dark matter*’ revealing tissue‐specific information

**DOI:** 10.1002/jev2.12481

**Published:** 2024-08-15

**Authors:** Navneet Dogra, Tzu‐Yi Chen, Edgar Gonzalez‐Kozlova, Rebecca Miceli, Carlos Cordon‐Cardo, Ashutosh K. Tewari, Bojan Losic, Gustavo Stolovitzky

**Affiliations:** ^1^ Department of Pathology Icahn School of Medicine at Mount Sinai New York USA; ^2^ Genetics and Genomic Sciences Icahn School of Medicine at Mount Sinai New York USA; ^3^ Icahn Genomics Institute Icahn School of Medicine at Mount Sinai New York USA; ^4^ Immunology and Immunotherapy Icahn School of Medicine at Mount Sinai New York USA; ^5^ Department of Urology Icahn School of Medicine at Mount Sinai New York USA; ^6^ DREAM Challenges

**Keywords:** biofluids, cancer, extracellular vesicles, liquid biopsy, RNA sequencing, small RNA

## Abstract

From eukaryotes to prokaryotes, all cells secrete extracellular vesicles (EVs) as part of their regular homeostasis, intercellular communication, and cargo disposal. Accumulating evidence suggests that small EVs carry functional small RNAs, potentially serving as extracellular messengers and liquid‐biopsy markers. Yet, the complete transcriptomic landscape of EV‐associated small RNAs during disease progression is poorly delineated due to critical limitations including the protocols used for sequencing, suboptimal alignment of short reads (20–50 nt), and uncharacterized genome annotations—often denoted as the ‘*dark matter*’ of the genome. In this study, we investigate the EV‐associated small unannotated RNAs that arise from endogenous genes and are part of the genomic ‘*dark matter*’, which may play a key emerging role in regulating gene expression and translational mechanisms. To address this, we created a distinct small RNAseq dataset from human prostate cancer & benign tissues, and EVs derived from blood (pre‐ & post‐prostatectomy), urine, and human prostate carcinoma epithelial cell line. We then developed an unsupervised data‐based bioinformatic pipeline that recognizes biologically relevant transcriptional signals irrespective of their genomic annotation. Using this approach, we discovered distinct EV‐RNA expression patterns emerging from the un‐annotated genomic regions (UGRs) of the transcriptomes associated with tissue‐specific phenotypes. We have named these novel EV‐associated small RNAs as ‘*EV‐UGRsʼ or “EV‐dark matter”*. Here, we demonstrate that EV‐UGR gene expressions are downregulated by ∼100 fold (FDR < 0.05) in the circulating serum EVs from aggressive prostate cancer subjects. Remarkably, these EV‐UGRs expression signatures were regained (upregulated) after radical prostatectomy in the same follow‐up patients. Finally, we developed a stem‐loop RT‐qPCR assay that validated prostate cancer‐specific EV‐UGRs for selective fluid‐based diagnostics. Overall, using an unsupervised data driven approach, we investigate the ‘*dark matter*’ of EV‐transcriptome and demonstrate that EV‐UGRs carry tissue‐specific Information that significantly alters pre‐ and post‐prostatectomy in the prostate cancer patients. Although further validation in randomized clinical trials is required, this new class of EV‐RNAs hold promise in liquid‐biopsy by avoiding highly invasive biopsy procedures in prostate cancer.

## 1 INTRODUCTION

Extracellular vesicles (EVs) are membrane‐enclosed entities released by cells into their microenvironment and systemic circulation (Chen et al., 2022; Horstman et al., 2007; Johnstone et al., 1987; Skog et al., 2008; Soleymani et al., 2023; Trams et al., 1981; Valadi et al., 2007). Recent evidence shows that EVs encapsulate small (∼13–200 nucleotides) functional RNAs (Bellingham et al., 2012; Crescitelli et al., 2021; Lässer et al., 2011; Skog et al., 2008; Valadi et al., 2007), which can serve as extracellular messengers and induce recipient cells to change their behaviour (Krishn et al., 2020; Lässer et al., 2011; Sharma et al., 2019; Valadi et al., 2007). Consequently, EVs and their RNA molecules are being recognized as a ‘messenger of the cellʼ—a message written in nucleic acids (Bellingham et al., 2012; Eldh et al., 2012; Kalluri & LeBleu, 2020; Nakamya et al., 2022; Valadi et al., 2007). These findings have resulted in major large‐scale transcriptomic efforts (such as the NIH funded extracellular RNA communication consortium (ERCC)) to delineate the extracellular RNA (exRNA) from various cells, disease types, and biofluids (Das et al., 2019; Lucien et al., 2023; Murillo et al., 2019; Rozowsky et al., 2019; Srinivasan et al., 2019; Wei et al., 2017). Subsequently, a large subset of EV‐associated small RNAs (such as miRNA, tRNA, siRNA, snRNA, YRNA, snoRNA etc.) have been reported as potential biomarkers (Chand et al., 2021; Chen et al., 2023; Gaglani et al., 2021), therapeutic mediators (Dogra & Stolovitzky, 2023; Lässer et al., 2011; Lee et al., 2018; Li et al., 2023), and post‐transcriptional regulators of gene regulation (Aday et al., 2021; Chen et al., 2018; Gaglani et al., 2021; Li et al., 2023).

The human genome comprises ∼2% of the well annotated protein coding exons (Harrow et al., 2012; Lander et al., 2001). However, majority of the RNA is transcribed from outside of the exons and protein‐coding regions (Harrow et al., 2012; Johnson et al., 2005; Kapranov et al., 2010). These findings manifest the genomic complexity and present new paradigms of disease specificity and genomic regulation via un‐annotated genomic regions (UGRs). Consequently, the UGRs, whose function and nomenclature remains poorly delineated, are denoted as the ‘*dark matter*’ of the genome (Johnson et al., 2005; van Bakel et al., 2010). Recent findings suggest that many RNA molecules which remain unannotated may play a critical role in regulation of gene expression (Bozgeyik, 2022; van Bakel et al., 2010). Yet, the current RNA transcriptomic strategies apply a conventional reference‐based approach, which is limited by their genomic annotations (Johnson et al., 2005). Consequently, if the biologically relevant signals arise outside the regions of the genomic annotations, then they would be neglected during the bioinformatic analysis (Bozgeyik, 2022). However, with major advances in next‐generation sequencing technologies (Hallal et al., 2020; Srinivasan et al., 2019) and novel computational tools (Abrams et al., 2019; Bellingham et al., 2012; Joo et al., 2019; Lu et al., 2018; Ritchie et al., 2015; Robinson et al., 2011), it is now possible to study the complete transcriptome, providing an unparallel opportunity to assess not only the annotated genomic regions (AGRs) but also the largely uncharacterized UGRs. In this context, EVs carry small (∼13–200 nt) coding and non‐coding RNAs (Chen et al., 2022). Furthermore, recent transcriptomic efforts show that many EV‐associated small RNAs arise from the non‐coding, intergenic/intronic genomic regions (Johnson et al., 2005; Kapranov et al., 2010; Vojtech et al., 2014). These important discoveries raise an important question, do EV‐associated small un‐annotated RNAs ‘*dark matter*’ have implications in disease regulation and specificity?

**FIGURE 1:**
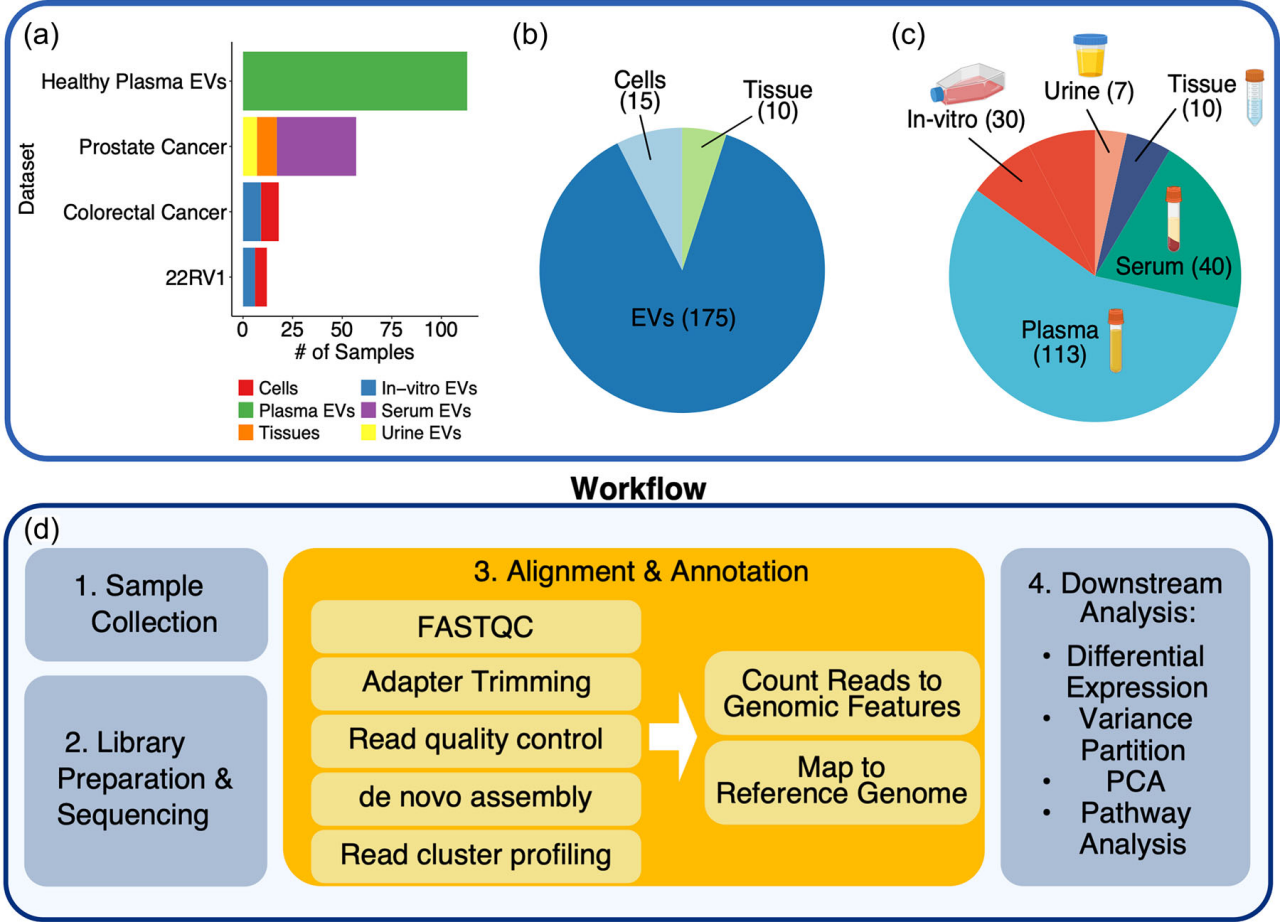
Overview of experimental design and data analysis. (A) Number of samples and subtype composition of each dataset included in the analysis. (B) Sources of sample types. (C) Biofluid profiles of all samples. (D) Workflow of the EV‐UGR analyses.

In this study, we investigate the EV‐associated small unannotated RNAs (EV‐UGRs) that arise from endogenous genes and are part of the genomic ‘*dark matter*’, which may play a key emerging role in regulating gene expression and translational mechanisms. To address this, we created a distinct small RNAseq dataset, followed by unsupervised data‐based pipeline and strategy that recognizes biologically relevant transcriptional signals irrespective of their genomic annotation. Using this approach, we identified distinct EV‐RNA expression patterns within UGRs, which are typically ignored during the conventional reference‐based annotation approach (Dobin et al., 2013; Frankish et al., 2019). Upon further investigation, we found that EVs carry distinct UGRs associated with tissue‐specific phenotypes. We show that EV‐UGRs gene expression is downregulated by ∼100 folds (FDR < 0.05) in the circulating serum EVs from aggressive prostate cancer subjects and is regained (upregulated) after prostatectomy, in the same follow‐up patients. Next, we asked if EV‐UGRs are restricted to inhouse datasets or can be recognized in external datasets. To address this, we investigated EV transcriptome in independent datasets including in‐house (cell lines, prostate tissue, serum EVs, urine EVs, total *n* = 62) and external exRNA datasets (plasma EVs, *n* = 113 and cell lines and EVs, *n* = 18) and confirmed EV derived UGR and AGR expression profiles in cells and EVs (datasets described in Figure 1A–D, Figure S1). Finally, we selected top EV‐UGRs, developed a custom stem‐loop RT‐qPCR assay, and validated their differential expression across different biofluids and tissue. Overall, we present an unsupervised data‐driven approach that identifies biologically relevant transcriptional signals even when there is no genomic annotation. Using this approach, we demonstrate that EV‐UGR signatures are novel small RNA candidates that carry tissue‐specific RNA associated with advanced prostate cancer.

## 2 METHODS

### 2.1 Isolation of blood serum EVs using nanoDLD

The EVs were isolated using these methods for results described in Figure 4. Before running biological samples, the integrated nanoDLD chips were first primed with bovine serum albumin, BSA. A F‐100 microtight connector with a 1/16″ OD, ∼2–4 cm long tube was fitted to the inlet port on the flow cell for this priming step. Additionally, a 1 mL syringe was prepared with a solution of 5% w/v BSA, (Sigma Aldrich) in a 1X phosphate buffer saline (Sigma Aldrich), the solution having been previously 0.02 µm filtered (Whatman). The syringe was attached to the tubing via a Luer Lock adapter. The excess DI water was then removed from the bump and zigzag outlet ports. The BSA syringe was set on the syringe pump and run at a fixed injection rate of 8 µm min^−1^ for *G* = 225 nm (*P*_app_ ∼ 5 bar). For the first 5 min, the bump outlet is sealed with a microplug to ensure all drain TSVs are cleared of residual bubbles, then after 5 min the bump is left unsealed so fluid flows through both ports. After priming with BSA, a 1 mL syringe with the sample fluid (human serum) was attached to the inlet tubing. For the *G* = 225 nm devices used, samples were 0.2 µm filtered. The tubing was purged, as in the bead case, until sample fluid exited the secondary port. The outlet ports were then cleared of any collected fluid, and the chip was allowed to run at 8 µL min^−1^. At this point, the sample run time clock was initiated, allowing sample to accumulate for 60 min. For the zigzag fluid, a F‐100 microtight was fitted to the zigzag outlet port with a short piece of tubing (∼5 cm), the end of which was inserted into a 1.5 mL, tared centrifuge tube sealed with parafilm to collect zigzag fluid as it exited the device. Zigzag fluid was allowed to continuously collect for the entire run time. For the bump outlet, fluid was removed every 30 min manually, using a pipet, and collected in a separate, tared tube. Samples were weighed to determine the volume collected from the bump and zigzag reservoirs and then stored in a 4°C fridge for analysis, typically within 24 h. EV isolation protocol with minor changes has also been published (Chen et al., 2022; Murillo et al., 2019; Smith et al., 2018; von Felden et al., 2021).

### 2.2 EV characterization with transmission electron microscopy (TEM)

After differential ultracentrifugation, the PBS‐resuspended isolate was evaluated with transmission electron microscopy (TEM) in a Hitachi 7000 transmission electron microscope operating at 80 kV. Briefly, equal volumes of the isolate and 3% Glutaraldehyde were mixed and kept at room temperature for 1 h. About 2 µL of osmium tetroxide was added to the mixture and incubated at room temperature for 1 h. The solution was then transferred to formvar coated TEM grids and observed under the electron microscope.

### 2.3 Immuno‐gold labeling of EVs

Frozen EVs pellet was brought to room temperature. Equal volumes of EVs and 3% glutaraldehyde were mixed and kept at room temperature for 1 h. About 2 uL of EV pellet was transferred to formvar coated TEM grid (at least two grids were prepared for each sample). TEM grids were covered and dried at room temperature for 30 min. 100 uL drops of PBS were transferred to Parafilm. Dried TEM grids were carefully washed by transferring them on top of the PBS drops with the help of forceps (this step is repeated five times). The grid is transferred to a 100 ul drop of BSA (this step is repeated five times). Then, the grid is transferred on top of a 5 uL drop of primary antibody (CD81) in a blocking buffer for 30 min. Transfer the grid to a washing buffer/blocking buffer for 5 min (this step is repeated five times). A drop of 5 uL gold conjugated secondary antibody is transferred to the Parafilm. The grid is transferred (and covered) on top of the gold antibody drop for 30 min. Once completely incubated, the grid is washed by keeping on top of 100 uL PBS solution for 3 min (this step is repeated 10 times). Finally, contrast the EVs on the TEM grid with osmium tetraoxide for 10 min. The grid is ready for TEM imaging.

### 2.4 Nanoparticle tracking analysis (size and zeta potential)

To estimate the size and concentration of the isolate, we conducted nanoparticle tracking analysis (NTA) samples were diluted to approximately 10^6^–10^7^ particles/mL in Millipore DI water. Particle concentration, size, and zeta potential were measured using ZetaView (Particle Metrix). Particle size and concentration were measured using the built‐in EMV protocol and zeta potential was measured using the built‐in EMV Zeta protocol.

### 2.5 Immuno‐fluorescence co‐localization analyses (Nanoview)

Briefly, canonical tetraspanin exosome markers CD81, CD9, and CD63 against the EV surface are arrayed on silicon chips. EV suspensions are incubated with the chips overnight. After incubation, chips are washed with PBS on a shaker and air dried. Captured EVs are detected using Single Particle Interferometric Reflectance Imaging Sensor technology.

### 2.6 RNA extraction, small library preparation and next‐generation sequencing

Total RNA was extracted from the serum bump fraction of nanoDLD, serum EVs pellet from UC using the total EV RNA and Protein Isolation Kit (Invitrogen 4478545). 50 µL EVs from bump fraction, 100–200 uL serum EVs were resuspended in equal volume of ice‐cold EV resuspension buffer. 2X denaturing solution was added to the final EVs solution on ice. Equal volume of acid‐phenol:chloroform solution was added to each sample. The final solution was vortexed for 60 s and centrifuged at 10,000 x *g*. The top aqueous phase was carefully isolated without disturbing the lower organic phase. The top aqueous phase was transferred to the provided filter cartridge in collection tubes. Bound RNA was washed three times using the included wash solution. Finally, a preheated elution solution was used to elute the RNA in 100 µL volume. RNA was stored at −20°C until it's RNA quality was assessed by bioanalyzer (Agilent 2100 Bioanalyzer, RNA 6000 Pico & small RNA Kit, Agilent Technologies).

cDNA Libraries were prepared for small RNAs using the SMARTer smRNA‐seq Kit for Illumina (Takara Bio 635030). A total of 18 cycles of PCR were carried out to obtain a good yield of cDNA from tissue, cells, and EVs. Final library quality was verified with Qbit and bioanalyzer. Negative (no RNA) and positive controls provided expected results. Next‐generation RNA sequencing was performed using a Novaseq (Illumina), 100 base pair, single‐end reads at the Mount Sinai Genomics core.

### 2.7 Quantification and statistical analysis

#### 2.7.1 Genome mapping

For quantification of gene expression, raw reads were aligned to the latest Ensembl GRCh38.p13 (GCA_000001405.28) using bowtie aligner (version 2.5.4b). FeatureCounts was then used to map the aligned reads to the GENCODE v26 primary gene annotation, including transcripts corresponding to ncRNAs such as lncRNA, miRNA as well as protein‐coding RNA. To maximize recovery and minimize the noise, multimapping reads were quantified up to *m* = 10 and distributed using unique reads mapping distribution, as described in most recent best practices protocols.

#### 2.7.2 Formal analysis

Data cleaning, filtering, and analysis were performed in R and under expressed genes or proteins with low or no counts across all samples of the similar phenotype were removed (at least one of the samples have CPM > 10). Normalization via trimmed mean of M‐values in edgeR ensures library sizes of all samples are scaled properly to minimize the influences of external factors. The limma package, originally designed for microarray data, performs linear modelling on normally distributed data. Thus, to accommodate for the non independent mean‐variance relationship of RNA‐seq data, the voom function assigns a precision weight derived from the library size and normalization factor of each sample itself to convert the raw counts to log2‐CPM values. The log2‐transformed counts minimize the changes in variance as the count size increases. Prior to examining differential expressions, we performed unsupervised clustering of samples to evaluate the similarities and dissimilarities between samples as well as across phenotypes of interest using the prcomp package in R. The result is reflected in the PCA plots. Differentially expressed genes are discerned between (1) cell lines versus cell‐line‐derived EVs, and (2) Prostate cancer patient serum‐derived EVs isolated using nanoDLD versus the EVs isolated using UC via the standard differential expression pipeline as illustrated in limma/edgeR packages. Results of the differentially expressed genes are represented in high‐resolution heatmap as well as volcano plots made using pheatmap and ggplot2 packages.

#### 2.7.3 Correlation analyses

Spearman Rho correlations were determined across cellular and EV genetic profiles as well as the proteomic profiles. Gene expressions were plotted in the *x*/*y* axis, where *x*/*y* axis are log2 (CPM), all RNA types were analysed.

#### 2.7.4 Biotype analysis

The gene biotype was recovered from the GTF annotation file for Ensembl GRCh38 (same as for alignment). Mapping resolution was kept as CDS with intron and exon annotation levels and combined to gene level when necessary. After differential expression quantification of gene biotype proportions, numbers and expression levels was taken into account. Thus, expressing gene biotype as (1) number of molecules per biotype (after lib. size adjustment) and (2) levels of expression using RPKM to adjust for gene/transcript length sizes.

#### 2.7.5 Characterization of UGRs

We identified UGR) using a moving‐window average approach. First, a minimum distance between UGRs was set at 75 base pairs, reflecting the typical trimmed read length from our sequencing approach. Second, an expression threshold was established, requiring a minimum of 100 reads per UGR. These criteria were determined based on the minimum read coverage of annotated small RNA read clusters. The rationale for these choices is visually depicted in Figure 4A. This allowed us to capture genome‐wide expression of clusters of RNAs in terms of UGRs, which typically overlapped known annotated RNA species (Figure 4A). We defined three characterization metrics for UGRs: (1) the *complexity* of UGRs as a read tiling efficiency or coverage entropy measure, defined on a continuous scale of 0 (single single‐read peak) to 1 (uniform distribution of reads); (2) peak coverage, the total number of reads supporting a UGR peak; (3) consensus sequence of the UGR peak. The set of all UGRs is the set of all accumulation loci of small RNA genes and their peak‐coverage consensus sequences, constituting average de‐novo assembled small RNA landscape with a standard count matrix. Overall, the UGRs have these general properties when combining both cellular and EV UGRs as a single set: (1) UGR length range between 10 and 10 kbp with a mean length of ∼674 bp (Figure S1B); (2) The complexity of UGRs reveals peak‐domination rather than uniform tiling (Figures 1C and 4); (3) The number of unique reads per cluster and the number of reads in the peak divided by the total UGR length shows values of 30% (Figure 4D). More importantly, UGRs capture previously unreported uncharacterized regions in the genome (Figure 4E–G). Interestingly, the number of unique sequences detected in UGRs are smaller in unannotated regions due their smaller size and higher frequency of multimapped reads (Figure 4F). Of note, UGR complexity further reveals that unannotated sequences are dominated by low complexity regions (Figure 4G). Finally, we explored the gene biotype composition of annotated UGRs and show that the RNA landscape is composed of highly expressing noncoding RNAs, pseudogenes, VDJ variable regions, miRNAs, rRNAs, mtRNAs and protein coding genes (Figure 4H). Finally, visually investigation of the expression patterns from EV‐RNA using Integrative Genome Viewer (Figure S1).

### 2.8 Trimming and mapping

The SMARTerTM smRNA‐Seq kit yields reads are flanked on the 5′ end by a leading triad of three bases from SMARTerTM template switching activity, and on the 3′ end by the Illumina adapter and extra bases from the oligo dT (which are exactly 15 bp in length). We used Cutadapt33 to remove the first three nucleotides of all reads, specify the homopolymer adapter sequence AAAAAAAAAA to remove along with any sequence 3′ of it, and finally discard all reads that are smaller than 15 bp long after these filters are applied. The exact command used, as recommended by the (strand‐sensitive) SMARTerTM smRNA‐Seq kit, is cutadapt‐m 15‐u 3‐a AAAAAAAAAA input.fastq>output.fastq. Therefore, our set of initial small RNAs are at least 15 bp long and are trimmed from positions 1–3 and also from the oligo dT 3′ through to the adapter. Next, we performed alignment using bowtie ‐n1 m15 allowing multimapping reads up to 15 sites producing bam files containing all alignments. Then, we use multimap rescue to recover multimapping reads with up to 15 multimapping sites. Finally, these resulting alignments are clustered using a distance of at least 75 pb between clusters. Briefly, all analysis were done on R studio platform using R Version 3.5 and several packages including limma, edgeR, ggplot2, reshape, tidyverse, variancePartition, pheatmap, annotables, GenomicFeatures, GenomicRanges and doParallel.

### 2.9 Construction of UGRs

The UGRs are constructed based on two factors: (1) A minimum distance between the reads, and (2) an expression threshold or minimum number of reads. These parameters were set at 75 bp and 100 reads based on the trimmed read length from sequencing and an arbitrary limit for what we consider a positive detection of RNA amplification (Figure 3A, Figure S1).

### 2.10 Profiling of UGRs using differential expression, correlations, and non‐parametric statistical tests

Here, we performed principal component analysis to evaluate the relationship between each sample UGR profile and variance analysis to determine the effect of demographics and technical variables in our experiments. Next, we used limma mixed linear models to perform differential expression analysis to estimate the significant (FDR < 5%) UGRs between EVs, cells, biofluids (serum, urine) and tissues (tumour and adjacent normal). To investigate whether exRNA and cellular UGRs capture (enclose) any key known RNA biotypes, we compared all genomic locus positions from UGRs and compared them to biotypes available in the ENSEMBL annotation for human genome version 38. The results were annotated and used for further comparisons between the samples. Two additional datasets were added as proof of principle for the properties of UGRs. The colorectal cancer and healthy patients samples, with cellular and exosomal parts were available for small RNAs. These samples were downloaded from https://exrna‐atlas.org/ and analysed through the same pipeline as our prostate cancer cohort. Next, we compared the results between differential expression between cells and EVs between prostate cancer and colorectal cancer cohort using spearman correlation and non‐parametric Wilcoxon rank test. Finally, we compared UGR properties between all three datasets and the respective biotype overlaps between them using the statistical methods described above.

### 2.11 Alignment to non‐human species and microbiome

To ensure that our EV‐UGRs are not a contribution from microbiome‐derived EVs, we use a prefiltering strategy to remove the microbiome contribution in our samples. We use a prefiltering strategy that consists of removing known sequencing artifacts, low quality reads and non‐human sequences. This step removes the microbiome contribution in our samples. Prefiltering is a way of addressing the issue by removing those genes that are unlikely to be differentially expressed and so reduce the overall number of tests performed. We performed EV RNA alignment (pre‐ and post‐filtering) to the non‐human species (including microbiome). To this end, we aligned our EV transcriptome with microbiome database and found that ∼10% of the EV transcriptome aligns with microbiome. We discovered that there is some contribution from the microbiome pre‐filtering however only less than 1% of microbiome contributed post filtering. The post filtering data is used for EV‐UGR detection in our study. The Figure S6 alignment of EV‐RNA to non‐human species and microbiome.

### 2.12 XDEC RNA‐Seq data deconvolution

To deconvolute RNA‐Seq data, we employed the XDec deconvolution algorithm, as implemented in the R script available at the following repository: XDec GitHub Repository. This method, developed by Oscar Murillo (odmurill@bcm.edu), is specifically designed for the deconvolution of complex RNA‐Seq datasets, enabling the extraction of cargo type composition from bulk RNA‐Seq data.

The following steps were undertaken using version 1.0.2 of the XDec algorithm (dated 2019‐03‐31):

Data preparation: Raw RNA‐Seq data was preprocessed to generate count matrices. Quality control checks were performed to ensure data integrity and eliminate low‐quality reads.
Normalization: The count matrices were normalized to account for differences in sequencing depth and other technical variations. Normalization was performed using established R packages to generate input suitable for the XDec algorithm.
Running XDec algorithm: The XDec R script was executed according to the instructions provided in the repository. Key parameters were set to default values, and the script was run in an R environment (version 3.6.3 or later). The execution of the script involved the following sub‐steps:
Loading the RNA‐Seq count matrices.
Defining the reference profiles for known cargo types, which were obtained from XDEC github.
Applying the deconvolution algorithm to estimate the proportions of different cargo types present in the bulk RNA‐Seq samples.
Post‐processing and validation: The output from the XDec algorithm, which includes estimated cargo‐type proportions, was further analysed. Validation of the deconvolution results was performed by comparing estimated proportions against known benchmarks from XDEC.
Statistical analysis: To ensure robustness, statistical analyses were conducted on the deconvoluted data to assess the accuracy and reliability of the estimated cargo type proportions. This involved correlation analyses, principal component analysis (PCA), and other relevant statistical tests.

By utilizing the XDec deconvolution algorithm, we were able to gain insights into the cargo type composition of the RNA‐Seq samples, thereby enhancing our understanding of the underlying biological processes and contributing to the overall goals of our study. The script and its comprehensive documentation facilitated the seamless application of this advanced deconvolution method to our data.

### 2.13 Proteomics

Each frozen pellet was homogenized by adding a pre‐determined volume of lysis buffer (2% SDS/1X protease inhibitor/0.1 M Ambic). Enhanced BCA Protein Quantification assay was used to determine the total protein amount from each sample. Three technical replications were run per sample. Proteins from 20ul of EV lysates were separated from SDS using micro S‐trap columns (http://www.protifi.com/s‐trap/) and digested on column by trypsin. Resulting peptides were speedvac dried for LC–MS/MS analysis. Thermo Orbitrap Fusion Tribrid Mass Spectrometer was used for MS/MS analysis. Global normalization based on total number of ms/ms spectra (PSM) acquired was applied to the MS data. Spectral counts were used for semi‐quantitative analysis to compare protein abundance among different samples.

Proteome Discoverer software (version 1.4) was used to search the acquired MS/MS data against a human protein database downloaded from the UniProt website. Positive identification was set at 5% protein FDR and 1% peptide FDR. Also, at least two unique spectra has to be identified per protein. Scaffold Proteome Software was used for post‐database search processing. 422 proteins passed the filtering criteria and their expression profiles among these four samples were analysed to identify differentially expressed proteins. Qlucore Omics Explorer Statistical Software was used to perform appropriate statistical analysis.

### 2.14 Vesiclepedia venn diagram

The open‐access Functional Enrichment Analysis Tool (FunRich) Version 3.1.3 was downloaded from http://microvesicles.org/#. Within this tool, the most commonly reported extracellular vesicle protein list was downloaded. Using the Venn Diagram tool in FunRich, plots were generated comparing the number of overlapping proteins identified in our experimental proteomics data from UC, UCDG, and DLD with the most commonly reported EV protein list. The percent overlapping proteins was calculated for experimental data UC, UCDG, and DLD, respectively.

## 3 RESULTS

### 3.1 Characterization of extracellular vesicles (EVs)

**FIGURE 2:**
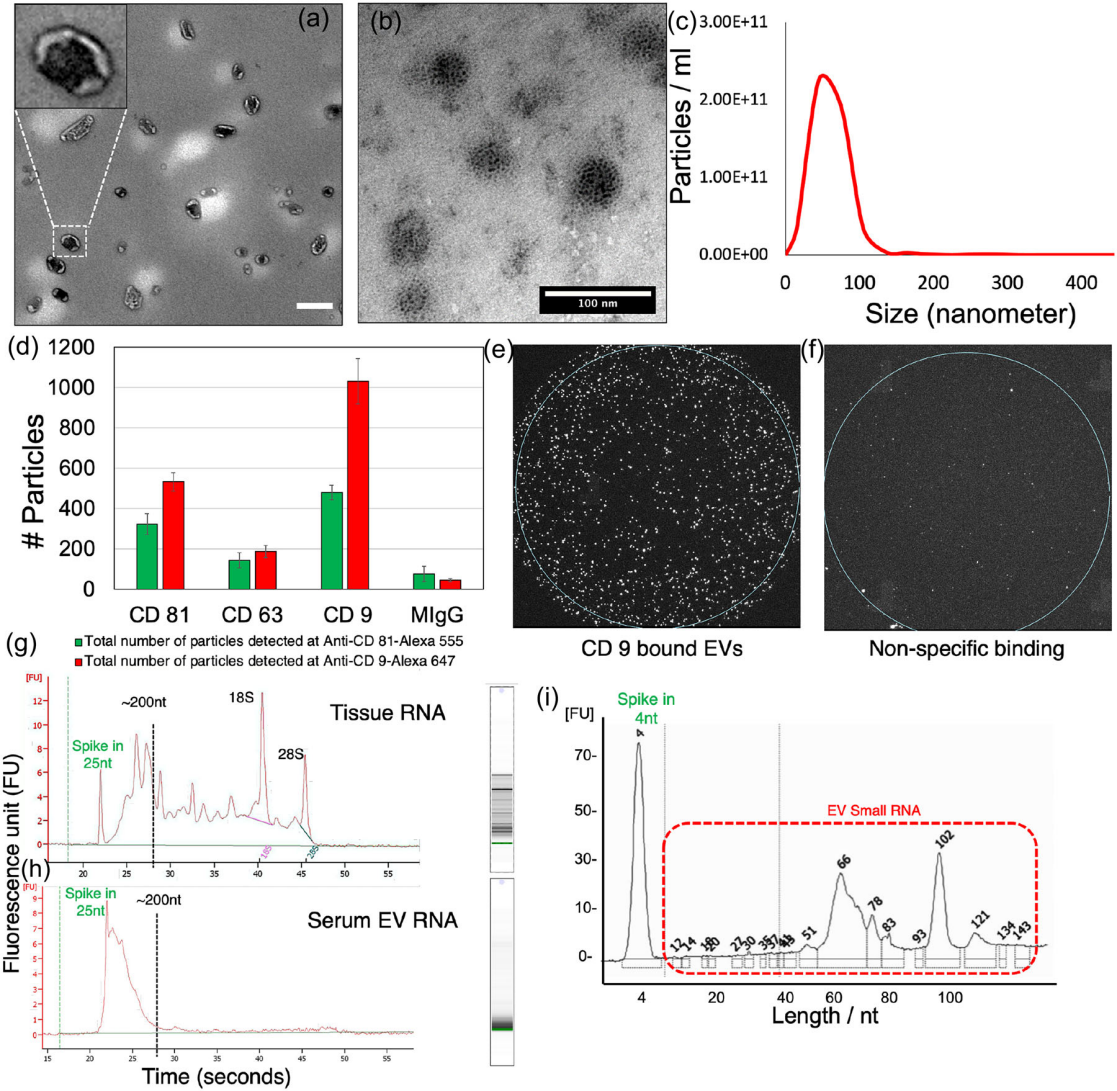
Characterization of the small EVs and their respective RNA cargo. (A) transmission electron microscopy (TEM) analyses of blood serum EVs from prostate cancer subjects. Scale bar is 100 nm. (B) Immunogold TEM analyses shows 6 nm gold‐CD81 (a canonical marker for EVs) on the surface of vesicles. (C) Nanoparticle tracking analyses (NTA) present particles between the size of ∼30–150 nm in diameter. Overall, ∼2 × 10^11^ particles/mL were detected in blood serum. (D) Immunofluorescence co‐localization study (nanoview) for canonical tetraspanins show EVs are enriched with CD9, CD63, and CD81. (E) Immunofluorescence co‐localization study shows EVs bound to the CD9 functionalized surface. (F) Minimum EV binding was observed when CD9 was not presented on the surface. (G & H) Capillary electrophoresis using pico chip for Bioanalyzer (Agilent) analyses revealed the size of small RNA in cells and EVs. (I) Capillary electrophoresis using small RNA chip for Bioanalyzer analyses revealed the size and distribution of small RNA in EVs.

In order to accurately characterize the EVs isolated in our study, we followed the minimal information for studies of extracellular vesicles (MISEV) (Lötvall et al., 2014; Théry et al., 2018; Welsh et al., 2024) guidelines—a field‐consensus rigor initiative of the International Society for Extracellular Vesicles (ISEV). Here, the EVs were observed under a high‐resolution transmission electron microscopy (TEM), revealing a frequently reported (Lässer et al., 2011; Li et al., 2023) round cup shaped morphology of ∼30–150 nm particles (Figure 2A, Figure S2A–C). Immuno‐gold TEM targeted to the small EV marker CD81 antibody attached to 6 nm colloidal gold particles displayed distribution of tetraspanin on the EV surface (Figure 2B). Nanoparticle tracking analyses (NTA) presented particles (∼2 × 10^11^particles/mL) between the size range of ∼30–150 nm in diameter (Figures 2C, S2A–C). Next, we applied multi‐colour immuno‐fluorescence co‐localization (Daaboul et al., 2016) tool known as the ExoView Tetraspanin Kit, a protein microarray chip with antibodies against commonly expressed small EV tetraspanin proteins CD9, CD63 and CD81. We quantified small EVs functionalized on immuno‐stained chips to confirm the presence of canonical small EV markers CD9/63/81 on their surface (Figure 2D & E). Figure 2E shows small EVs functionalized on CD9 immune‐stained chips and negligible non‐specific binding, when no tetraspanins were functionalized the substrate (Figure 2F). Taken together, small EVs isolated in our study display round, cup shaped morphology, of ∼30–150 nm particles in diameter and displayed enrichment of canonical small EV tetraspanin markers (CD9/63/81) with minimum non‐specific binding on the immuno‐stained substrate. Furthermore, our recent publication conducted comprehensive characterization of EVs’ proteo‐transcriptome in separate studies (Chen et al., 2022; Li et al., 2023). Next, we characterized the RNA from prostate tissue and patient matched serum derived EVs using capillary electrophoresis (Figure 2G & H). We noticed that while the prostate tissue displayed small RNA and large ribosomal RNAs (18S and 28S) (Figure 2G), the small EVs only enriched small RNA of <200 nucleotides (nt) (Figure 2H). We confirmed these results using another analogous small RNA chip that allows for higher resolution separation of small RNAs ranging below ∼200 nt, displaying two major peaks at ∼66 nt and ∼102 nt (Figure 2I). Moreover, recent studies have confirmed that large RNAs (greater than ∼3000 bp) are depleted in small EVs but are present in their donor cells and macrovesicles (Lässer et al., 2011; Valadi et al., 2007; Wei et al., 2017). Overall, these results suggest that the RNA cargo of tissue and EVs are distinct. Tissues mostly displayed large ribosomal RNA (rRNA) (18S and 28S) and small RNA below ∼200 nt, and small EVs did not display rRNA and enriched with small RNA below ∼200 nt (Figure 2G & H). Within the small RNA landscape, EV‐RNA displayed two major peaks at ∼66 nt and ∼102 nt (Figure 2I), providing preliminary evidence of the different subtypes of small RNA. To address the different subtypes of proteins and RNAs in EVs, we proceeded with proteomics and total small RNA sequencing (discussed below).

### 3.2 Proteomic and exRNA cargo type analyses of extracellular vesicles

A common confounder of EV RNA and exRNA studies is that RNA in complex biological samples such as blood serum is associated with several extracellular particles, such as lipoproteins, Argonaute2 (AGO2), and diverse EVs (Arroyo et al., 2011; Turchinovich et al., 2011). For this reason, it is important to assess the removal of non‐EV RNA carriers such as lipoproteins (Driedonks et al., 2018; Nolte‐’t Hoen et al., 2012). To address this concern, we took two different approaches: (1) A proteomic analyses of EVs isolated using different technologies, followed by their comparison of MISEV 2023 recommended proteins (EV hallmarks and commonly co‐isolated non‐vesicular extracellular particles (NVEPs)) with our datasets; (2) We utilized ERCC developed exRNA XDEC toolkit to address the RNA cargo proportions from AGO2, lipoproteins, and small EVs in our isolations. Below we discuss these approaches.

**FIGURE 3:**
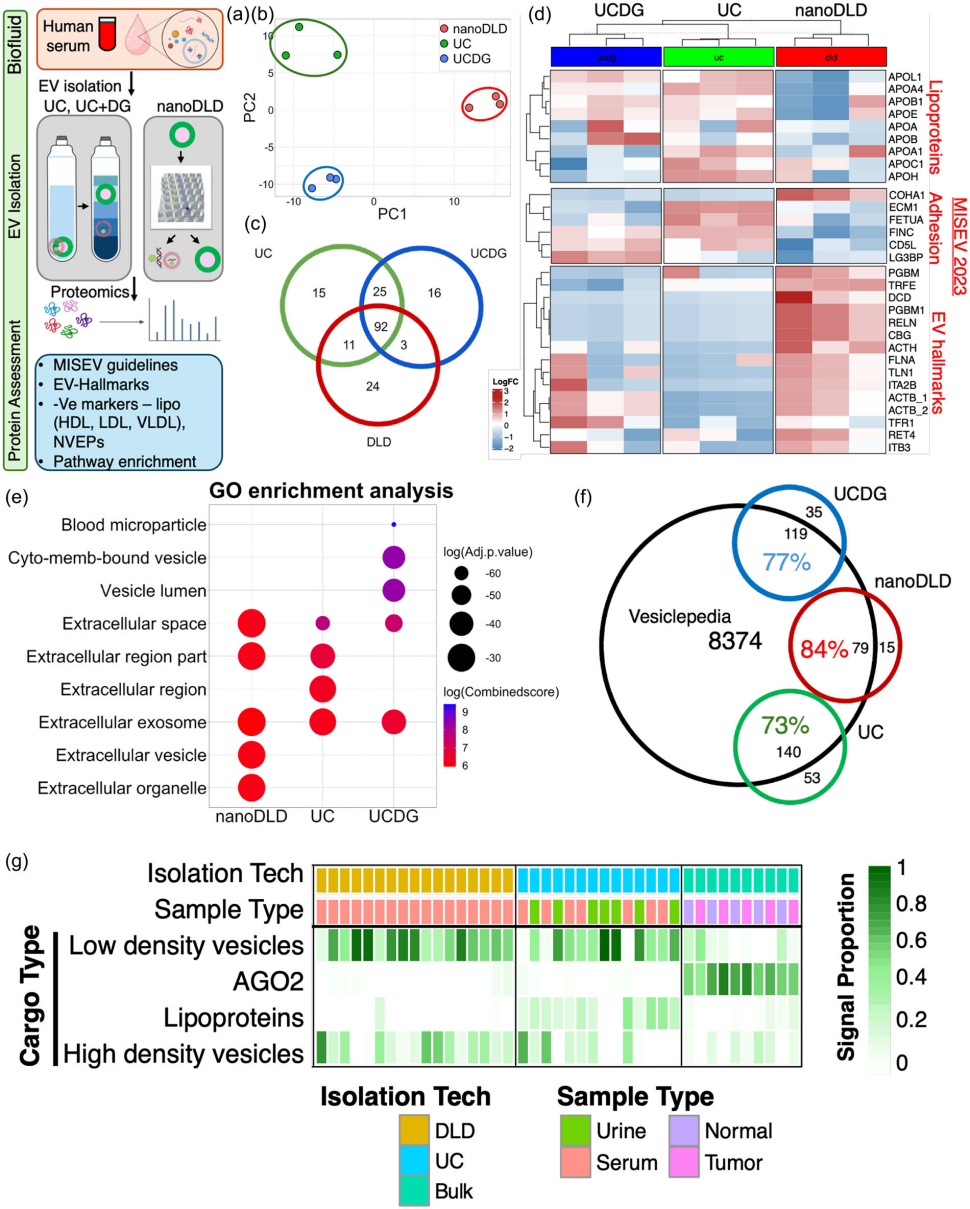
Mass spectrometry and exRNA cargo type analyses of EVs (A) Workflow: Serum EV isolation using different platforms: ultracentrifugation (UC), UC in conjunction with density gradient (UC+DG), and nanoDLD, followed by proteomics and EV‐hallmark and pathway enrichment analyses. We isolated EVs from three different human subjects and compared between three different isolation methods (total *n* = 9). (B) Principal components analysis (PCA) to identify possible batch effects between technologies. (C) Venn diagram comparison of common and unique EV proteins among three isolation technologies. (D) MISEV 2023 recommended EV, NVEP, and EVP hallmark analyses. Heatmap includes proteins from MISEV 2023 (Welsh, et al., JEV 2023, Table 3. Protein content‐based EV characterization) recommended EV‐hallmarks for EVs and NVEPs markers. Lipoproteins, and other common co‐isolates, and EV‐proteins are presented. E) Gene ontology pathway analyses is performed, and top pathway enrichment scores are compared. All three technologies used for EV isolation demonstrated ontologies‐based pathways of Extracellular exosome. nanoDLD EVs were enriched with ontologies related to Extracellular vesicle, Extracellular organelle, Extracellular exosome (Figure 3E, Table S2). UC and UC+DG also displayed the presence of blood microparticle pathway ontology (due to lipoprotein contribution). Notably, the blood microparticles associated ontology was absent in the nanoDLD EVs proteome. (F) The top enriched proteins from our proteomics datasets are compared with Vesiclepedia database. (G) exRNA Atlas cargo type analyses to delineate cargo type proportions for EVs from Serum, urine, and tissue samples (bulk) are compared. Overall, the EVs isolated in our study enrich with high and low density vesicles associated RNA with minimum contribution from AGO2 and lipoprotein‐associated RNA. Total samples used for this study: nanoDLD (*n* = 17), UC (*n* = 14, urine = 7, serum = 7), bulk tissue samples (*n* = 10, tumor = 5, normal = 5.

1. Proteomics approach to delineate EV cargo: To uncover the ubiquitous and abundant EV proteins, we used an unbiased and quantitative proteomic approach of liquid chromatography coupled to high‐resolution mass spectrometry. We have conducted these analyses on EVs isolated using ultracentrifugation (UC), UC in conjunction with density gradient (UC+DG), and nanoDLD (Figure 3A). To ensure the reproducibility of proteomic analyses, we isolated EVs from 3 different human subjects and compared from three different methods (total *n* = 9). Principal components analysis (PCA) identified distinct clustering of EV‐proteome by their method of isolation, demonstrating possible batch effects between the technologies (Figure 3B). Venn diagram comparison uncovered that ∼82% of the nanoDLD proteins were common with UC and UC+DG, while enriching for ∼18% unique proteins (Figure 3C). We then carefully followed the MISEV 2023 guidelines (Table 3. Protein content‐based EV characterization) (Welsh et al., 2024). We compared the curated list of MISEV 2023 recommended proteins (EV hallmarks and commonly co‐isolated NVEPs) with our datasets (Figure 3D). A high resolution heatmap shows that the lipoproteins (ApoA, ApoB, ApoC, ApoE, ApoH, and ApoL), which are commonly associated with NVEPs (such as HDL, LDL, vLDL, and Chylomicrons) were consistently depleted in the proteome of nanoDLD isolated EVs. Notably, the nanoDLD isolated EVs were enriched with MISEV recommended proteins (Welsh et al., 2024), such as integrins (ITGA, ITGB, talin (TLN1)), transferrin receptor (TFR), heparan sulphate proteoglycans (PGBM, PGBM1, RELN), ESCRT protein (TRFE), and extracellular matrix proteins (Dermcidin/DCD, Filamin/FLNA, RET4). All three technologies used for EV isolation demonstrated ontologies‐based pathways of Extracellular exosome (Figure 3E). nanoDLD EVs were enriched with ontologies related to Extracellular vesicle, Extracellular organelle, Extracellular exosome (Figure 3E). In contrast, UC and UC+DG also displayed presence of some blood microparticle pathway ontology (due to lipoprotein contribution). Notably, the blood microparticles associated ontology was absent in the nanoDLD EVs proteome. The cohort of depleted proteins in nanoDLD EVs was predominantly composed of lipoproteins and nuclear proteins. UC and UC+DG also displayed depletion of nuclear proteins and some lipoproteins. We did not detect many EV co‐isolates (often used as negative controls for EVs) such as AGO2 proteins, calnexins (CANX), and Golgi matrix (GM130) protein in our EV samples from all three technologies. Finally, we utilized the large EV proteomic datasets from the Vesiclepedia database (a compilation of data from over ∼1300 EV studies) (Kalra et al., 2012). A comparison of our EV proteomic data with the Vesiclepedia database displayed an overlap of ∼84% of the EVs proteins in our study relative to those in the Vesiclepedia (Figure 3F). UC and UC+DG also showed an overlap (with Vesiclepedia) of 73% and 77%, respectively (Figure 3F). These results confirmed that our EV isolation methods and proteomic analyses were consistent, relatively reproducible, and reliable compared to the other external studies present in the Vesiclepedia database and as per MISEV guidelines.
2. exRNA analyses to delineate EV cargo: A recent exRNA transcriptomic investigation from ERCC (Murillo et al., 2019) investigated and compared extracellular RNA cargo types in ∼5000 human samples using different isolation methods and biofluids. This independent analysis presented RNA cargo types associated with lipoproteins (HDL, LDL, and VLDL), argonaute proteins, and high & low‐density vesicles (Murillo et al., 2019). In this study, we utilized ERCC developed exRNA XDEC toolkit to address the RNA cargo proportions from AGO2, lipoproteins, and small EVs in our isolations (Figure 3G). Total samples used for these analyses are: nanoDLD (*n* = 17), UC (*n* = 14, urine = 7, serum = 7), bulk tissue samples (*n* = 10, tumour = 5, normal = 5). Using the XDEC toolkit, we discovered that nanoDLD EVs enrich for RNA associated with high and low density vesicles (Figure 3G). Notably, nanoDLD EVs did not show enrichment of AGO2 and lipoprotein‐associated RNA (Figure 3G). Similarly, UC EVs showed enrichment of RNA from high/low density vesicles with minimum contamination from lipoproteins. The RNA cargo type analyses for bulk prostate tissue showed relatively high enrichment of RNA associated with AGO2 proteins. Overall, the EVs isolated in our study enrich high and low density vesicles associated RNA with minimum contamination from AGO2 and lipoprotein‐associated RNA. In contrast, prostate tissue displayed relatively high AGO2‐associated RNA, confirming that AGO2 and lipoproteins‐associated RNA are enriched in the cells and depleted in our EV isolation.

Taken together, we have conducted a comprehensive proteomic and exRNA analyses of EVs presented in this study. We demonstrate that nanoDLD isolated EVs are enriched with EV‐associated, MISEV recommended protein, displayed ontologies of Extracellular exosome and Extracellular vesicle, and depleted lipoproteins. The cohort of depleted proteins in EVs was Argonaute2 (AGO2), calnexins (CANX), Golgi matrix (GM130), and nuclear proteins in our EV samples from all three technologies. Finally, to address the exRNA cargo type proportions of AGO proteins, lipoproteins, and small EVs in our isolations, we used ERCC developed exRNA XDEC toolkit. We found that nanoDLD EVs carry high and low density vesicles associated RNA with minimum contamination from AGO2 and lipoprotein‐associated RNA. The fact that nanoDLD is able to deplete lipoproteins with great precision are reassuring primarily because nanoDLD is precisely designed as a microfluidic device that can sort, enrich ∼100 nm particles, and remove large > 200 nm (microfluidic channel width is 200 nm), and <50 nm (including HDL, LDL, and AGO) with >90% accuracy. These finding have been reported in recent peer reviewed publications (Chen et al., 2022; Dogra & Stolovitzky, 2023; Kim et al., 2017; Murillo et al., 2019; Smith et al., 2018; Wunsch et al., 2016). Figure S3A–D shows an overview of nanoDLD chip technology, where ‘Bumpʼ fraction sorts ∼100 nm polystyrene particles, while ‘zigzagʼ fraction contains ∼25 nm polystyrene particles. Of note, ∼25 nm beads do not concentrate with but ∼95 nm beads concentrate ∼35 folds with nanoDLD chip technology.

### 3.3 Small RNA transcriptomic analyses reveal distinct un‐annotated EV‐RNAs

**FIGURE 4:**
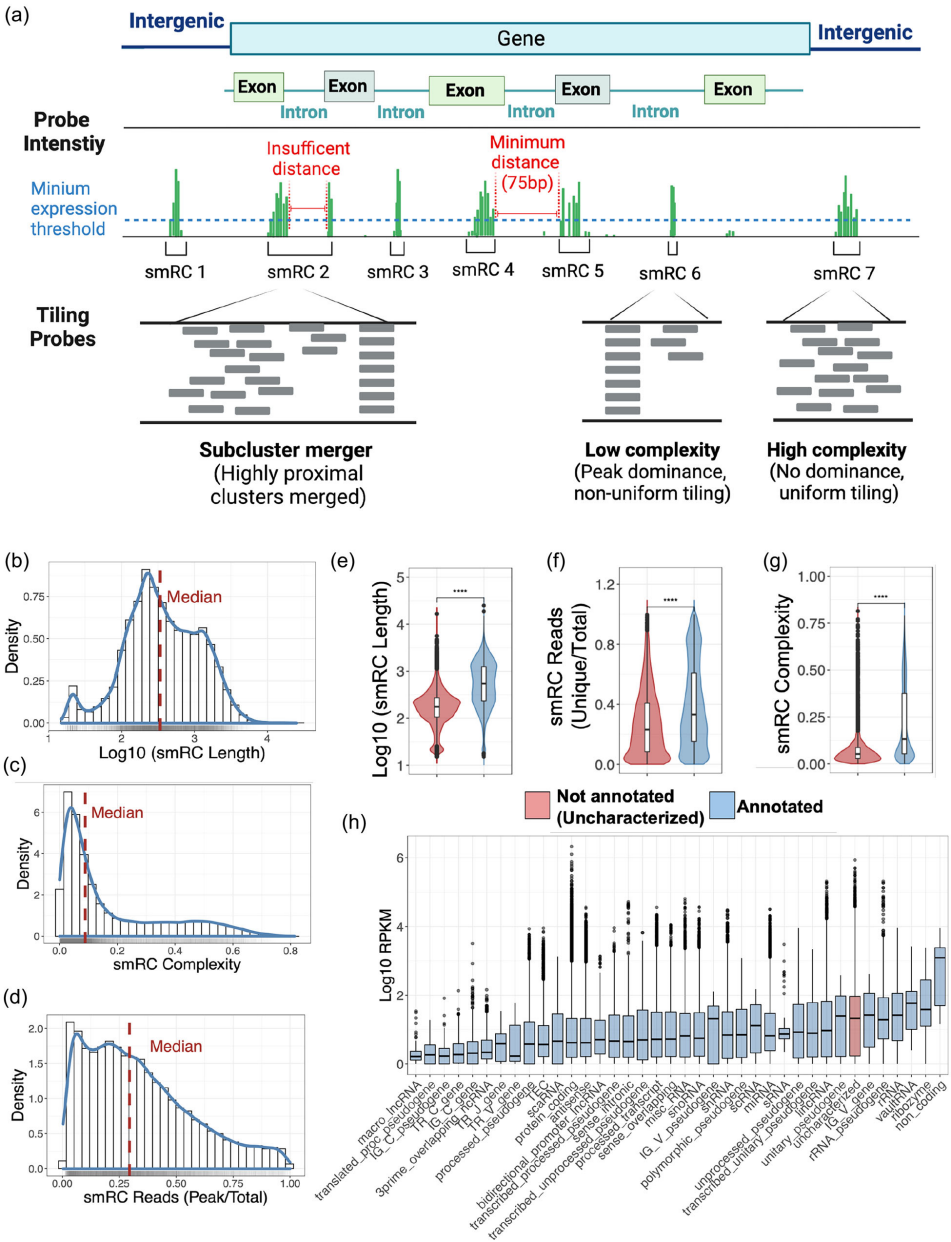
Characterization of UGRs/small read clusters (smRCs). (A) Schematic drawing of reads aligning on the genome highlighting 3 representative examples that help define UGRs and their properties. Left image or UGR/smRC2, has a minimum expression threshold representative of at least 100 reads aligned in a tight genomic region. This region is represented by a read pile up and very few surrounding reads besides the peak. Thus, showcasing a peak dominant UGR without uniform tiling or low complexity. Central image or UGR/smRC2 represents a region without any peaks or no peak dominance with a uniform tiling composition or high complexity. Additionally, between smRC1 and smRC2 there is a minimum distance of 75 base pairs arbitrarily defined by the read size of our experiment that separates these two clusters. Right image or UGR/smRC7 represents a region with insufficient distance between a peak and a uniform tiling. Thus, both regions composed by the uniform tiling and the peak constitute the UGR 3, of moderate complexity. (B) The length of a UGR in our dataset varies from 14 to 10000 base pairs shows a function of density of all detected UGRs. (C) The distribution of complexity values for prostate cancer UGRs, has a median ∼0.1 highlighting that the majority of UGRs are of low complexity. (D) Density function of the number of reads in the peak of a UGR divided by the total number of reads in the same UGR, shows a median of ∼0.3. (E, F) The difference in length between annotated (blue) and unannotated UGRs (red) with statistical significance (FDR < 0.05) computed with wilcoxon rank test. Additionally, Uncharacterized, or not annotated UGRs (red) also have lower number of unique reads and lower complexity. (G) compared to annotated regions (blue). (H) The annotated UGRs correspond not only to protein genes but to a wide spectrum of different genomic elements such as pseudogenes, lincRNAs, snoRNAs, rRNAs and other non‐coding RNAs shown in blue. Additionally, the expression of uncharacterized UGRs (red) is also higher than well studied miRNAs (blue) or protein coding genes (blue).

Recently, EV‐associated RNA transcriptomics have gained widespread interest for understanding their cargo, communication potential, and liquid‐biopsy biomarkers (Bellingham et al., 2012; Chen et al., 2022; Murillo et al., 2019; Smith et al., 2018). Yet, as of today, the complete (annotated and un‐annotated) transcriptomic landscape of EVs remains poorly understood (Murillo et al., 2019; Rozowsky et al., 2019; von Felden et al., 2021). Recent studies have identified critical limitations, including suboptimal alignment of short reads (20–50 nt) and missing genomic annotations (Murillo et al., 2019; von Felden et al., 2021). To address these challenges, novel approaches for EV‐transcriptomic analyses are needed, along with the urgent development of robust, reproducible, open‐access and user‐friendly protocols for EV diagnostics, quality control, and analysis. Here, we created an unsupervised data‐based strategy that recognizes biologically relevant transcriptional signals irrespective of their genomic annotations (Methods & Figures 4A, S4 & S5). To quantify the small RNA expression patterns, we used ‘*de novo*’ assembly and defined UGRs using the following criteria (Methods & Figure 4B–D): (1) A minimum distance between the reads, and (2) an expression threshold. The expression properties of UGRs were defined as small read cluster (smRC) length (Figure 4B), smRC complexity (Figure 4C), smRC number of reads (Figure 4D). The difference in length, complexity, and reads between annotated (blue) and unannotated UGRs (red) with statistical significance (FDR < 0.05) was computed with wilcoxon rank test (Figure 4E–G). Additionally, uncharacterized, or not annotated UGRs (red) also have lower number of unique reads and lower complexity compared to annotated regions (blue) (Figure 4G). The annotated UGRs correspond not only to protein genes but to a wide spectrum of different genomic elements such as pseudogenes, lincRNAs, snoRNAs, rRNAs and other non‐coding RNAs shown in blue (Figure 4H). Additionally, the expression of previously uncharacterized UGRs (red) is also higher (Figure 4H) than well studied miRNAs (blue) or protein coding genes (blue). Using this identification method, we were able to identify, quantify, and perform differential expression of UGRs expression profiles in human prostate cancer & benign tissues, and EVs derived from blood, urine, and cell culture media (discussed below). Finally, among the 38 different RNA biotypes that we investigated, UGRs were ranked 7th by expression (Figure 4H), indicating the prevalence of highly expressed UGRs signals, yet ignored during analyses. A list of all 128 identified UGRs loci by their genomic location is provided in Table S1.

### 3.4 Serum EV‐RNA expressions are altered pre‐ and post‐prostatectomy

**FIGURE 5:**
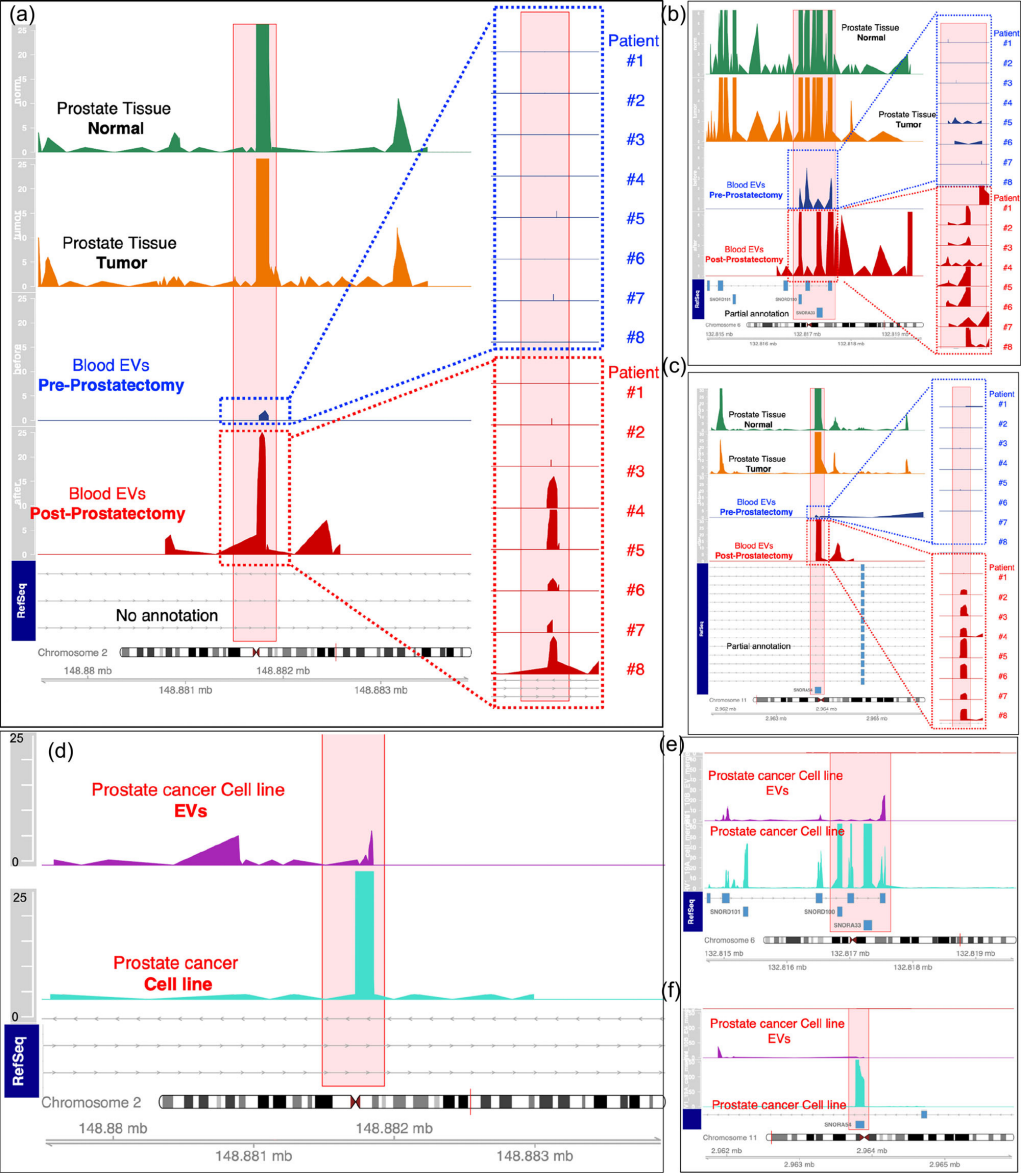
Visualization of selected UGRs in prostate cancer patients (tumor, normal, serum EVs pre‐ & post prostatectomy) and in human prostate carcinoma epithelial cell line (22RV1) and their EVs. Three distinct examples of UGRs identified at Chromosome 2, 6, and 11 visualized in Integrated Genome Browser. Expression patterns emanating from the un‐annotated (A) and partially annotated (B, C) genomic regions from Chromosome 2, 6, and 11, respectively. EV‐UGRs expression is downregulated pre‐prostatectomy and upregulated post‐prostatectomy in all three examples. Inset: UGR expression pattens from 8 prostate cancer (pre‐ and post‐prostatectomy) subjects. (D) Human prostate carcinoma epithelial cell line (22RV1) and their EVs also show similar expression patterns in un‐annotated (D) and partially annotated (E, F) genomic regions from Chromosome 2, 6, and 11, respectively. To ensure reproducibility, EVs are isolated using two different technologies nanoDLD (A–C) and UC (D–F). Reference sequence (Refseq) shows no annotation (A, D) or partial annotations (B, C, E, F) for the selected genomic regions.

Next, we visually investigated the expression patterns from EV‐RNAs (using Integrative Genome Viewer) (Robinson et al., 2011). These analyses helped us identify unique expression patterns stemming from the UGRs. We detected UGR expression patterns at several regions of the genome, including intergenic and intronic regions in different chromosomes. Importantly, we discovered that circulating EV‐UGRs have a significant association with tissue specific phenotypes, as demonstrated in the distinct example of transcriptional signal in chromosome 2 (Figure 5A). The UGR expression patterns were present in the prostate normal (Figure 5A, green) and tumour tissue (Figure 5A, orange). Next, we discovered that the serum EV‐UGRs were downregulated in the prostate cancer subjects pre‐prostatectomy (Figure 5A, blue panel labelled as ‘pre‐prostatectomyʼ, shows average expression from eight cancer subjects, inset shows individual expression from all eight subjects). Remarkably, the lost expression patterns re‐emerged once the same eight subjects underwent radical prostatectomy and ∼2–3 months after had undetectable PSA (Figure 5A, red panel). Similarly, chromosome 6 (Figure 5B) and chromosome 11 (Figure 5C), displayed UGRs expression patterns from only partially annotated intergenic and intronic regions. All three select EV‐UGRs were suppressed ‘pre‐prostatectomyʼ and re‐emerged ‘post‐prostatectomyʼ (Figure 5A–C). Notably, the prostate cancer subjects discussed above are the same matching subjects and their blood serum was collected pre‐prostatectomy and were followed up ∼2–3 months post‐prostatectomy. The EVs isolation, RNA isolation, and sequencing was performed on all samples at the same time. Overall, EV‐UGR signatures represent prostate cancer tissue‐specific information in serum, which is downregulated during cancer but is re‐emerged after prostatectomy in the matched cancer patients representing biologically relevant information. Of note, the human genome (Hg38) showed no or only partial annotation in regions of the UGR expression in human tissue, blood EVs, and in human prostate carcinoma epithelial cell line (22RV1) and their EVs (Figure 5A–F).

### 3.5 Prostate tissue and EVs derived from serum and urine carry distinct UGRs that span across the genome

**FIGURE 6:**
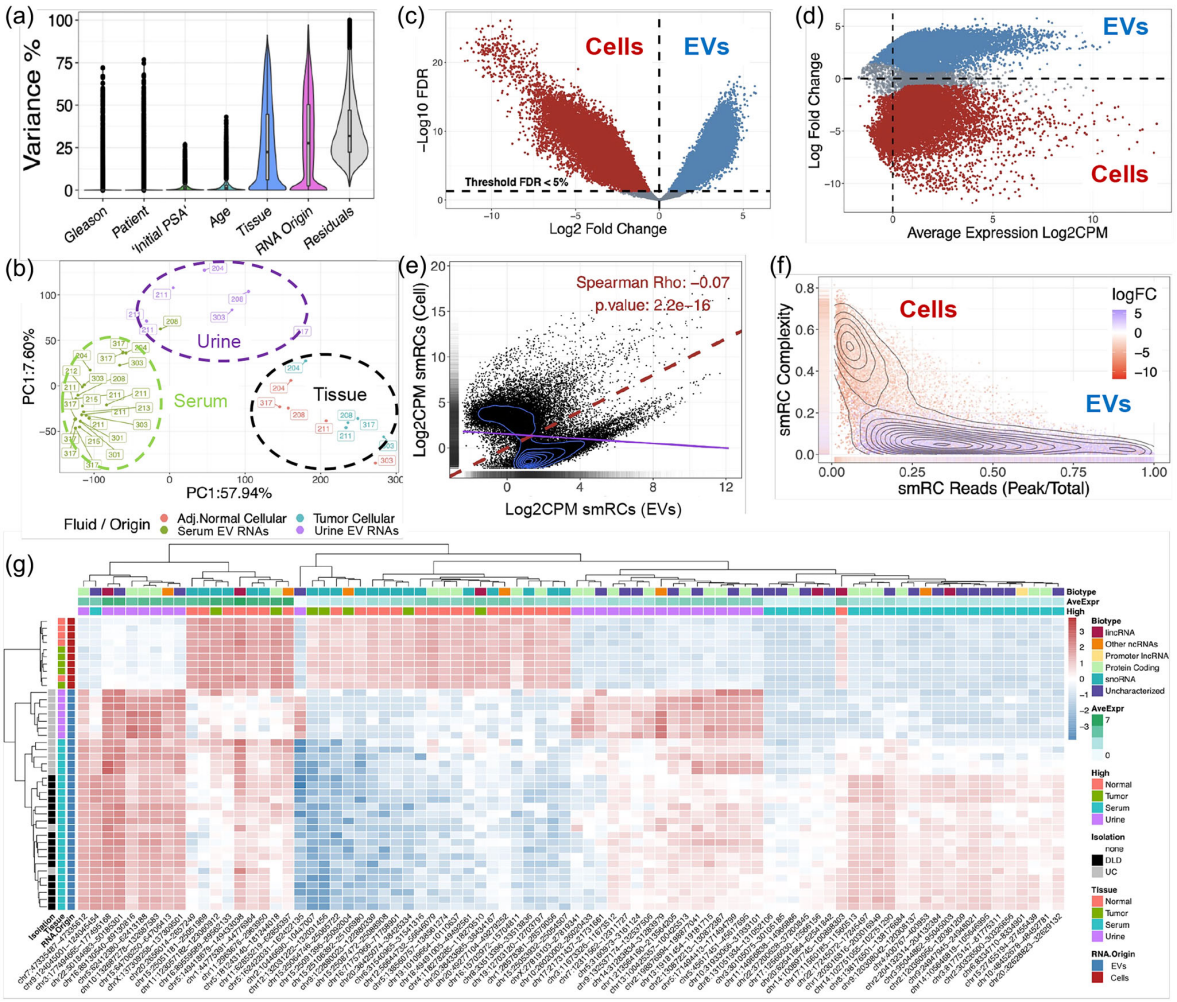
Cellular and EVs profiles reveal distinct new UGRs. (A) Variance partition analyses shows that origin of RNA is the main driver of variance in our data followed by age and PSA. (B) Principal component analysis (PCA) resulted in the complete separation of the samples based on origin of RNA by tissue, urine, or serum. (C) The differential expression between cellular and EVs UGRs colored by EVs (blue) or cell (red). (D) MAplot showing the average expression in log2CPM scale colored by EVs (blue) or cell (red). (E) Spearman correlation between cells and EVs in log2CPM scale showing no correlation between either UGR expression. (F) The complexity of UGRs is shown in the y axis while the percentage of reads in the peak compared to the total reads is shown in x axis. These properties of cells or EVs show that the centers of density (black lines) align completely with their differential expression profiles (colors red for cells and blue for EVs) indicating that UGR properties also are defined by their RNA origin. (G) A selection of top UGRs for tumor, adjacent normal, urine or serum are shown in a heatmap. The annotation for samples is shown in the columns on the left of the plot for isolation type, tissue and RNA origin. The annotation on top of the heatmap indicates the average expression levels, the differential expression status (high in either tumor, adjacent normal, serum or urine) and the gene biotype for each UGR.

To examine whether different variables (patient age, PSA, Gleason score, tissue type, biofluid, and RNA origin) influence the UGR expression patterns, we profiled the variance associated with phenotypic features using mixed effect models (Gonzalez‐Kozlova, 2022; Hoffman & Roussos, 2021; Ritchie et al., 2015). We found that the origin of RNA was the main driver of variance in our data followed by patient age and PSA (Figure 6A). Principal component analysis (PCA) resulted in the separation of the samples based on tissues, urine, and serum transcriptomes (Figure 6B). Our differential expression analysis showed unique expression of cellular profiles compared with EVs (Figure 6C & D). Next, we computed the spearman correlation between cellular and EV profiles, showing that EVs possess distinct (spearman rho = −0.07) enrichment of UGRs (Figure 6E, F). Finally, we present the top differentially expressed UGRs (in normal and tumor tissues, and EVs from serum and urine) that span across the genome and present in several chromosomes (Figure 6G).

**FIGURE 7:**
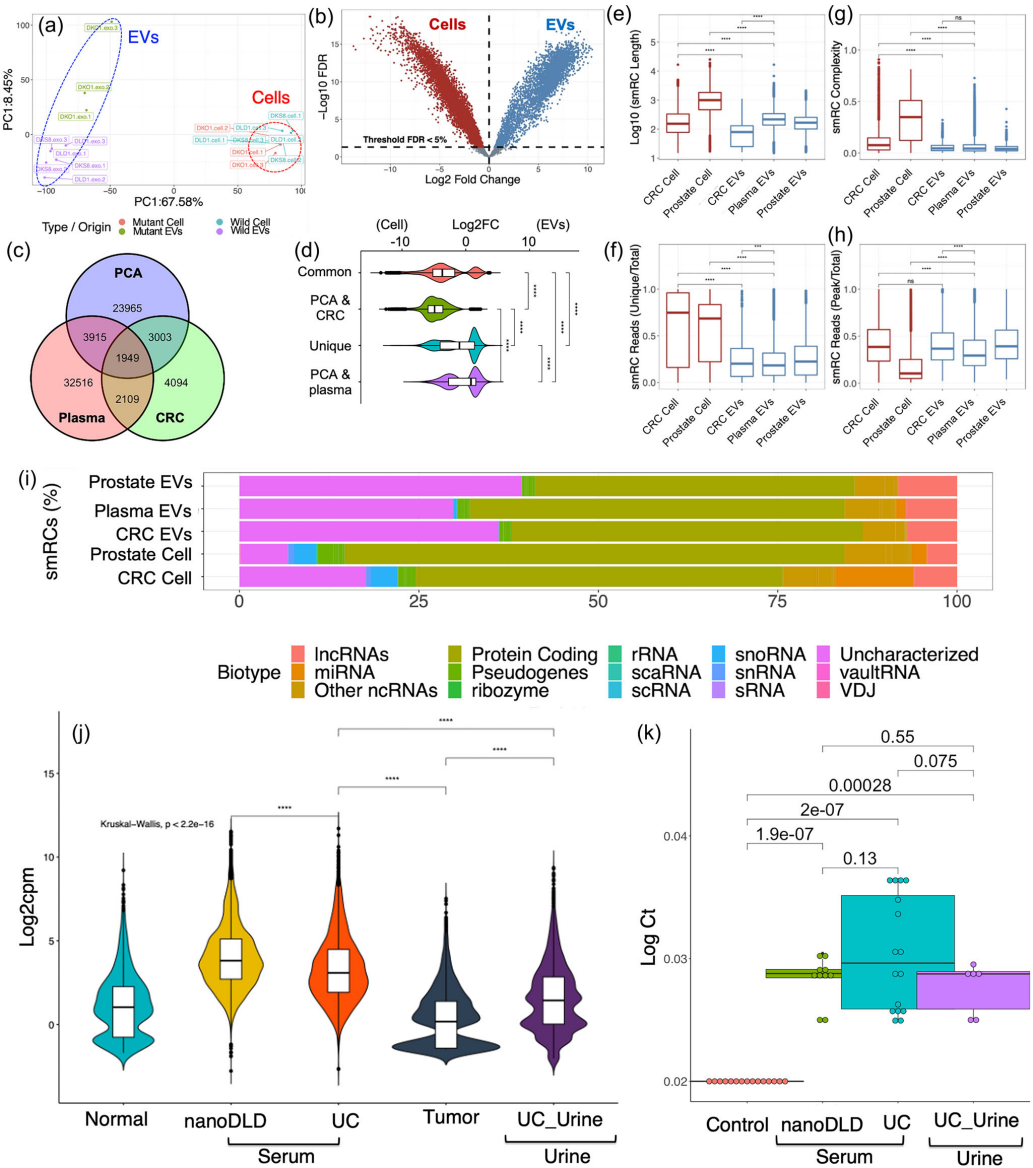
Identification of UGRs in independent external datasets and development of a simple RT‐qPCR assay. (A) Principal component profiles for CRC cell lines colored based on the wild or mutant (KRAS) status for either cells or EVs, showing a separation between each phenotype. (B) differential expression volcano plot between cells (red) and EVs (blue). (C) Overlap between UGRs from either cohort (Our PCA, EERC CRC cell lines and EERC health plasma patients. (D) The common UGRs between either cohort separated by their logFC based on cell/EVs comparison. (E) Comparison using Wilcoxon rank test between the lengths of UGRs from either cell (red) or EVs (blue) from different cohorts. (F) Comparison using Wilcoxon rank test between the percentage of unique reads from either cells (red) or EVs (blue). (G) Comparison using Wilcoxon rank test for the complexity values of either cells (red) or EVs (blue). (H) Comparison using Wilcoxon rank test for percentage of reads in the peak between cells (red) and EVs (blue). (I) Gene biotype composition between cells or EVs, showing that EVs contain a larger non coding and unannotated RNA footprint than cells. (J) RNAseq quantification of UGRs selected from differential expression pool. (K) RT‐qPCR validation of select EV‐UGRs from RNAseq data. No significant differences were found between technologies of EV isolation. Normal: normal tissue, Tumor: tumor tissue, Serum: serum EVs, Urine: urine EVs, Control: no template control. Following UGRs were tested for RT‐qPCR: chr2:148881489‐148881928, chr2:222918489‐222918711, chr8:21329709‐21329879.

Next, to validate our in‐house dataset and determine if UGRs are present in independent datasets, we used two NIH ERCC RNAseq datasets (Murillo et al., 2019) of in vitro colorectal cancer (CRC) cells and plasma derived EV RNA (Figure 7A). The PCA showed clear separation of UGR profiles across cellular, EV categories, normal and knockout (KO) cellular RNA (Figure 7A). Next, we performed differential expression of the cellular and EVs components in this dataset identifying the UGRs with FDR < 1% (Figure 7B). Then, we compared the overlap between all three datasets (Figure 7C), showing that each dataset contained not only unique but also ∼9000 shared UGRs (Figure 7C). Interestingly, cellular UGRs had a higher expression than those found in EVs (Figure 7D). Similar to the prostate cancer dataset described in Figure 6, in vitro CRC cellular UGRs were larger and contain a greater read proportion compared to EVs UGRs (FDR < 1%) (Figure 7E & F). Additionally, EVs UGRs are less complex and have a greater read peak/totals proportion than their cellular counterparts (Figure 7G & H). Finally, we investigated the composition of cellular and EV UGRs across our datasets, revealing that EV‐UGRs carry larger proportions of unannotated genomic composition of small RNA and represent ∼20%–40% of the whole transcriptome (Figure 7I).

### 3.6 EV‐UGRs can be used a biomarker

Most RNA‐based studies apply conventional reference based approach and thus are limited to the annotated genomic regions (Joo et al., 2019; Rozowsky et al., 2019; Ziemann et al., 2016). As a result, most extracellular RNAs used for liquid‐biopsy studies are annotated (Kapranov et al., 2010; van Bakel et al., 2010; von Felden et al., 2021). Here, to ascertain that EV‐UGRs presented in this study are not a biproduct of the library prep, alignment, or other computational biases, we selected top prostate tissue‐specific UGRs, carefully designed targeted primers, and orthogonally validated their differential expression in our patient cohort by RNAseq (Figure 7J) and developing a stem‐loop RT‐qPCR assay (Figure 7K). We then compared RT‐qPCR results with expression patterns from RNAseq data (Figure 7K). The RT‐qPCR results correlated with the RNAseq expression, discarding our hypothesis of alignment or other computational bias. RT‐qPCR validation of select EV‐UGRs from RNAseq data showed that no significant differences were found between technologies (nanoDLD and UC) of EV isolation (Figure 7K). The three select UGRs for RT‐qPCR validation were located in regions of chromosomes 2q, 8q (chr8:21329709‐21329879, chr2:148881489‐148881928, chr2:222918489‐222918711), and to the best of our knowledge, have not been studied before. Interestingly, some of the smRCs in our RNAseq analyses were selectively present in serum EVs but absent in the urine EVs (Figure 6G) (this finding will be pursued in our future studies). Although preliminary, our analyses suggest that EVs‐UGR RNAs may play a key emerging role in regulating gene expression, translational mechanisms, and can be used as biofluid‐based biomarkers for minimally invasive (serum) or non‐invasive (urine) diagnosis. Since, most previous studies have not investigated the ‘*dark matter*’ uncharacterized genomic regions, there is little comparable data with a complete mapping of RNA species from the EVs. Regardless, these early findings contain major fluid‐based diagnostic implications for many diseases, including cancers with precise measurements of up‐ or down‐regulated EV‐UGRs.

## 4 DISCUSSION

In this study, we investigate the EV‐associated small unannotated RNAs (EV‐UGRs) that arise from endogenous genes and are part of the genomic ‘*dark matter*’, which may play a key emerging role in regulating gene expression and translational mechanisms. To address this, we created a distinct small RNAseq dataset, unsupervised bioinformatic pipeline, and detected biologically relevant transcriptional signals outside of their genomic annotation. We found that EVs carry distinct UGRs associated with tissue‐specific phenotypes and that EV‐UGR gene expressions are downregulated by ∼100 fold (FDR < 0.05) in the circulating serum EVs pre‐prostatectomy from aggressive prostate cancer subjects. These select EV‐UGR expression signatures were regained (upregulated) post‐prostatectomy in the same follow‐up patients. Taken together, our study suggests that small unannotated RNAs that arise from endogenous genes and are part of the genomic ‘*dark matter*’, may play a key emerging role in regulating gene expression and translational mechanisms.

There are several possible explanations for the transcriptional activity that appears outside of annotations. These signals could be transcriptional ‘noise’, experimental artifacts, transposons, or previously undetected coding or noncoding genes (Bozgeyik, 2022; Johnson et al., 2005; Mcclintock, 1956; Rinke et al., 2013). The fact that ‘*dark matter*’ transcription has been observed by different groups (Bozgeyik, 2022; Johnson et al., 2005; Kapranov et al., 2010; Turchinovich et al., 2014; Vojtech et al., 2014), on different platforms (van Bakel et al., 2010), in different species (Huang et al., 2020; Kapranov et al., 2010; Rinke et al., 2013), with similar outcomes suggests artifacts are not the dominant cause (Bozgeyik, 2022; van Bakel et al., 2010). Furthermore, multiple lines of evidence suggest that more of the genome is transcribed than represented by current annotations (Johnson et al., 2005). Importantly Turchinovich et al. (2014) detected known gene loci, as well as unannotated loci and noted that ∼35% of unannotated RNA reads did align to the Human Microbiome Project database—indicating that there is exogenous RNA sequencing in human plasma. In the context of EV‐associated UGRs, Turchinovich (Huang et al., 2020; Turchinovich et al., 2014), Vojtech et al. (2014), and Huang et al. (2020), have reported unannotated loci. To ensure that our EV‐UGRs are not a contribution from microbiome‐derived EVs, we use a prefiltering strategy that consists of removing known sequencing artifacts, low quality reads and non‐human sequences (Figure S6) Alignment of EV‐RNA to non‐human species and microbiome). Alternatively, the UGR reads could have emerged due to the following reasons: (1) immature partial transcripts with introns not spliced out yet; (2) unannotated exons which might represent alternative splicing; (3) the repetitive regions or transposons are overexpressed, and such overpopulated regions need to be secreted out of the cell, which is something that EVs have been reported to conduct (Kutchy et al., 2020). Finally, the data presented here continues to reinforce the hypothesis that the transcriptional landscape of EVs is complex and it is clear that in‐depth biochemical experiments will be required to assess the biological relevance of ‘*dark matter*’ transcripts. In line with above hypotheses, if the noncoding RNAs were dismissed as a mere transcriptional noise, gene regulation would not have been delineated in its current form of understanding (Elbashir et al., 2001; Fire et al., 1998). A key remaining question is how do UGRs disseminate from cells to the EVs and what is their underlying function. Future studies along the lines described here will optimistically delineate their role in gene regulation and may enable noninvasive monitoring of cancer, neurodegenerative, and other disease biomarkers and identification of tissue specific UGRs RNA to potentially avoid unnecessary surgical procedures.

### 4.1 Study limitations

There are limitations in the study design that prevent overly broad conclusions. First, the sample number is relatively small to control for all types of potential biases. Second, limited previous studies have characterized UGRs (von Felden et al., 2021), therefore, there are limited comparable studies to date given the lack of complete mapping of RNA species from EVs as well as the characterization of UGRs in EVs. Third, the method used to characterize UGRs could differ across study groups, depending on their sequencing workflow and pipelines. However, this study stands as a demonstration of the applications and possibilities of utilizing ‘de novo’ assembly characterization for EV profiling to identify previously unannotated and novel RNA sequences. Indeed, we identified novel RNA sequences as potential novel biomarkers for prostate cancer. However, further validation and a larger cohort is needed to improve the robustness of the results presented here. Studies show that exRNA can be associated with EVs, AGO proteins, lipoproteins, and many other extracellular particles (Arroyo et al., 2011). In our studies, we observed RNA in both nanoDLD isolated ‘EV‐enriched’ and ‘EV‐depleted’ fractions (Figure S3A). nanoDLD is one of the better technologies to precisely sort small EVs (∼50–200 nm) and deplete larger >200 nm, and smaller <50 nm particles, including AGO and lipoproteins with >90% accuracy. Our analyses of nanoDLD EV proteomics (Figure 3A–F) and exRNA cargo type (Figure 3G) analyses reassured us that the cargo type in our study is high or low density vesicles with minimum RNA contribution from AGO2 and other lipoproteins. Finally, although we have compared three different EV isolation technologies to reduce variability, it has been well accepted that isolation method may contribute dramatically on downstream molecular components from isolated EVs. Thus, caution must be observed while using different technologies for EV isolation. Overall, these data and methods provide novel metrics that can be used in future experiments to monitor a wide spectrum of disease and healthy phenotypes and to help establish non‐invasive molecular tools for tracking the impact of unannotated and non‐coding RNAs.

### 4.2 Data and code availability

Protocols for EV separation and characterization are provided with this manuscript and will be made available on EV TRACK (http://evtrack.org/) upon publication of the manuscript. Data reported in this paper has been shared by the lead contact. This paper does not report original code. The code used is available on https://github.com/chentytina/22RV1_EV or upon request. RNA‐seq data is available at https://www.ncbi.nlm.nih.gov/geo/ can be searched with GEO number = GSE123736. Any additional information required to reanalyse the data reported in this paper is available from the lead contact upon request.

## AUTHOR CONTRIBUTIONS

**Navneet Dogra**: Conceptualization (lead); data curation (lead); formal analysis (equal); funding acquisition (equal); investigation (lead); methodology (lead); supervision (lead); visualization (equal); writing – original draft (lead). **Tzu‐Yi Chen**: Data curation (equal); formal analysis (equal); validation (equal); visualization (equal); writing – original draft (equal); writing – review and editing (equal). **Edgar Gonzalez‐Kozlova**: Data curation (equal); formal analysis (equal); methodology (equal); validation (equal); visualization (equal); writing – original draft (equal); writing – review and editing (equal). **Rebecca Miceli**: Writing – original draft (equal); writing – review and editing (equal). **Carlos Cordon‐Cardo**: Resources (equal); supervision (equal); writing – review and editing (equal). **Ashutosh K. Tewari**: Resources (equal); supervision (equal); writing – review and editing (equal). **Bojan Losic**: Formal analysis (equal); software (equal); visualization (equal). **Gustavo Stolovitzky**: Conceptualization (lead); funding acquisition (equal); investigation (equal); supervision (lead); writing – review and editing (equal).

## CONFLICT OF INTEREST STATEMENT

The authors declare no conflicts of interest.

## Supporting information


**Supplementary Figure 1**. (A) Details of participating PCa patients used in current study.
**Supplementary Figure 2**. TEM imaging, NTA analyses, and zeta potential analyses of patient serum (A), Urine (B), and cell line (22RV1) derived EVs.
**Supplementary Figure 3**. (A) Overview of nanoDLD chip technology.
**Supplementary Figure 4**. UGR identification pipeline.
**Supplementary Figure 5**. Visually investigation of the expression patterns from EV‐RNA using Integrative Genome Viewer.
**Supplementary Figure 6**. Alignment of EV‐RNA to non‐human species and microbiome.
**Supplementary Table 1**. List of identified UGRs loci by their genomic location.

Supporting Information

Supporting Information

Supporting Information

Supporting Information
