# Supplementary material for: Extracellular vesicles carry transcriptional ‘dark matter’ revealing tissue‐specific information
Source: J Extracell Vesicles. 2024 Aug 15;13(8):e12481. doi: 10.1002/jev2.12481 (PMC11327273; doi:10.1002/jev2.12481)
Supplement: Supplementary file 1 — Supplementary Figure 1. (A) Details of participating PCa patients used in current study. Supplementary Figure 2. TEM imaging, NTA analyses, and zeta potential analyses of patient serum (A), Urine (B), and cell line (22RV1) derived EVs. Supplementary Figure 3. (A) Overview of nanoDLD chip technology. Supplementary Figure 4. UGR identification pipeline. Supplementary Figure 5. Visually investigation of the expression patterns from EV‐RNA using Integrative Genome Viewer. Supplementary Figure 6. Alignment of EV‐RNA to non‐human species and microbiome. Supplementary Table 1. List of identified UGRs loci by their genomic location. [file JEV2-13-e12481-s004.pdf]

Extracellular Vesicles Carry Transcriptional '*Dark Matter*' Revealing Tissue-Specific Information.

Navneet Dogra[1,2,3,#,], Tzu-Yi Chen[1, #], Edgar Gonzalez-Kozlova[4,#], Rebecca Miceli[1], Carlos Cordon-Cardo[1], Ashutosh K. Tewari[5], Bojan Losic[2], Gustavo Stolovitzky[2,6,]**

Affiliations:

[1]Department of Pathology, Icahn School of Medicine at Mount Sinai, New York 10029, United States.

[2]Genetics and Genomic Sciences, Icahn School of Medicine at Mount Sinai, 10029, United States.

[3]Icahn Genomics Institute, Icahn School of Medicine at Mount Sinai, 10029, United States.

[4]Immunology and Immunotherapy, Icahn School of Medicine at Mount Sinai, New York 10029, United States.

[5]Department of Urology, Icahn School of Medicine at Mount Sinai, New York 10029, United States.

[6] DREAM Challenges

# These authors have contributed equally to the work.

* Correspondence:

1. Dr. Navneet Dogra

   Department of Pathology, Molecular and Cell-Based Medicine

   Icahn School of Medicine at Mount Sinai, New York 10029, United States.

   navneet.dogra@mssm.edu

2. Dr. Gustavo Stolovitzky

   Founder and Director, DREAM Challenges powered by Sage Bionetworks

   Adjunct Professor, Department of Biomedical Informatics

   Department of Systems Biology, Center for Computational Biology and Bioinformatics

   Columbia University

   gustavo.stolo@gmail.com

Supporting information

REAGENT or RESOURCE	SOURCE	IDENTIFIER
**Antibodies**		
CD81	Abcam	ab239687
Gold conjugated secondary antibody	Abcam	ab105285
**Biological Samples**		
Serum samples from prostate cancer patients	Icahn School of Medicine at Mount Sinai; Department of Urology	IRB, GCO # 06-0996, 14-0318
Human prostate cancer cell line 22RV1	American Type Culture Collection	ATCC CRL-2505
**Chemicals, Peptides, and Recombinant Proteins**		
RPMI 1640 cell culture medium	GIBCO	
Bovine serum albumin in phosphate buffer saline	Sigma Aldrich	
3% Glutaraldehyde	Sigma Aldrich	
Osmium tetraoxide	Sigma Aldrich	
Acid-phenol	Invitrogen	Cat# 4478545
Chloroform	Invitrogen	Cat# 4478545
Lysis Buffer (2% SDS/1X protease inhibitor/0.1M Ambic)	Invitrogen	Cat# 4478545
**Critical Commercial Assays**		
Total EV RNA and Protein Isolation Kit	Invitrogen	Cat# 4478545
**Deposited Data**		
Raw and normalized 22RV1 gene counts<br>Prostate cancer patients tumor, benign, blood serum, urine (n=41)	This manuscript	GSE123736<br>Provided with this manuscript.
Human reference genome Ensembl GRCh38.p13	Ensembl	https://useast.ensembl.org/Homo_sapiens/Info/Annotation
Human plasma EVs	Felden et al. 2021	PMID: 34321221
Human breast cancer cell lines EV and cell proteomic counts	Hurwitz et al., 2016	PMID: 27894104
Human colon cancer cell lines EV and cell gene counts	Hinger et al., 2018	PMID: 30332650
ERCC datasets	https://exrna.org	
**Software and Algorithms**		
QIAGEN Ingenuity Pathway Analysis	QIAGEN	https://digitalinsights.qiagen.com/products-overview/discovery-insights-portfolio/analysis-and-visualization/qiagen-ipa/?cmpid=QDI_GA_IPA&gclid=CjwKCAjwgr6TBhAGEiwA3aVuIS7UzgcUf8gTC8ad6uXMnBwZEeEb_lhG88SStmpLMASvAUl0tbI9tBoCJeAQAvD_BwE
FeatureCounts read summarization function	Subread	http://subread.sourceforge.net/
bowtie aligner (version 2.5.4b)	bowtie	http://bowtie-bio.sourceforge.net/bowtie2/index.shtml
R version 4.1.1	R Core Team	https://www.R-project.org/.
Qlucore Omics Explorer software	Qlucore	https://qlucore.com/
Code availability	Github	https://github.com/chentytina/22RV1_EV
**Resources and samples information.**		

A

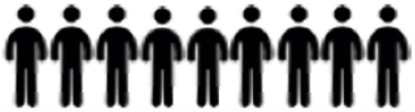

Patient	Age Range	G.S.	stage	PSA
P1	65-70	4+4	T2	12.56
P2	70-75	4+5	T3	2.7
P3	55-60	4+4	T3	19
P4	65-70	3+4	T2	7.95
P5	45-50	3+4	T2	7.1
P6	55-60	4+3	T2	10.5
P7	55-60	4+3	T2	5.1
P8	65-70	4+3	T2	46
P9	65-70	4+4	T2	4.9

B

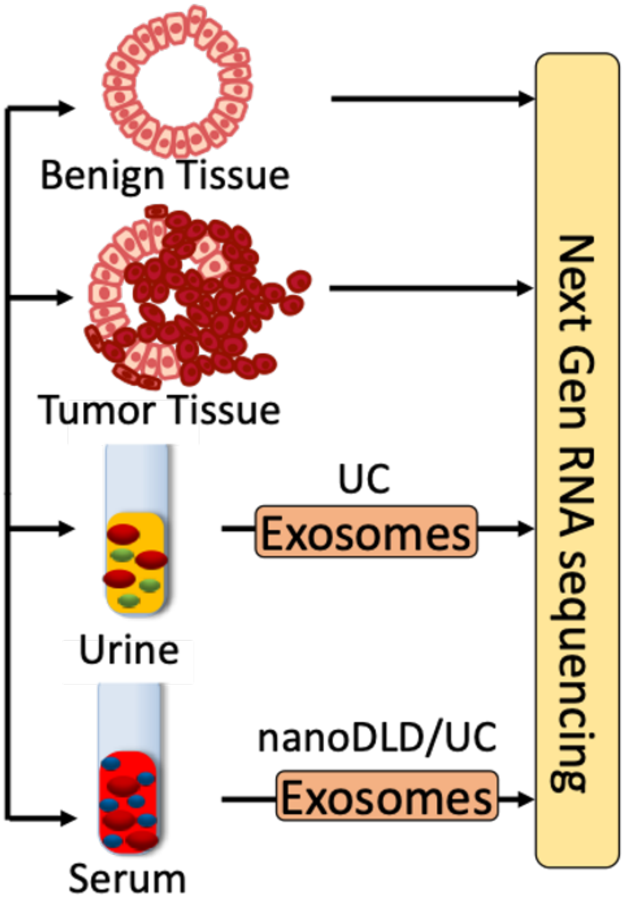

C

PATIENT	MONTHS PASSED SURGERY WHEN THE SAMPLE WAS COLLECTED	BX GLEASON	PATH GLEASON	INITIAL PSA	POST PSA
10	3-4	4+3	3+4	5.8	<0.006
11	<1	4+4	4+3	4.5	n/a
12	3-4	4+3	4+3	6	<0.006
13	~3	4+4	4+4	8.7	0.026
14	1-2	3+3	3+3	6.5	<0.006
15	4	3+4	3+4	10.5	<0.006
16	3-4	3+3	3+3	3.3	<0.006
17	4-5	3+3	3+3	4.5	<0.006

Supplementary Figure 1. (A) Details of participating PCa patients used in current study. A total of 17 prostate cancer patients were studied. All patient serum was used for EVs isolation through nanoDLD, while 5 patients' tissue (normal and tumor), urine and serum were also used for EV isolation through UC. (B) An overview for RNAseq study of PCa patient's Tissue and bodily fluids. G.S. – gleason score, PSA – prostate specific antigen. Bottom: Details of participating PCa patients monitored before and after prostatectomy. C) Eight patients returned

to clinic after prostatectomy and their EVs isolated again, and EV transcriptome was compared pre- and post-prostatectomy.

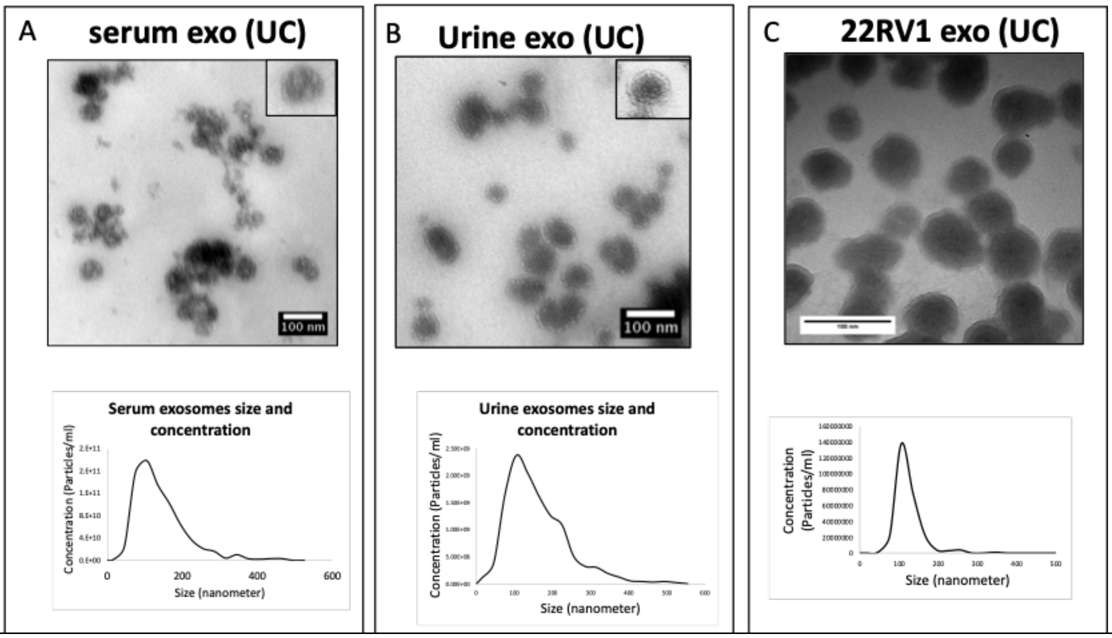

Supplementary Figure 2. TEM imaging, NTA analyses, and zeta potential analyses of patient serum (A), Urine (B), and cell line (22RV1) derived EVs.

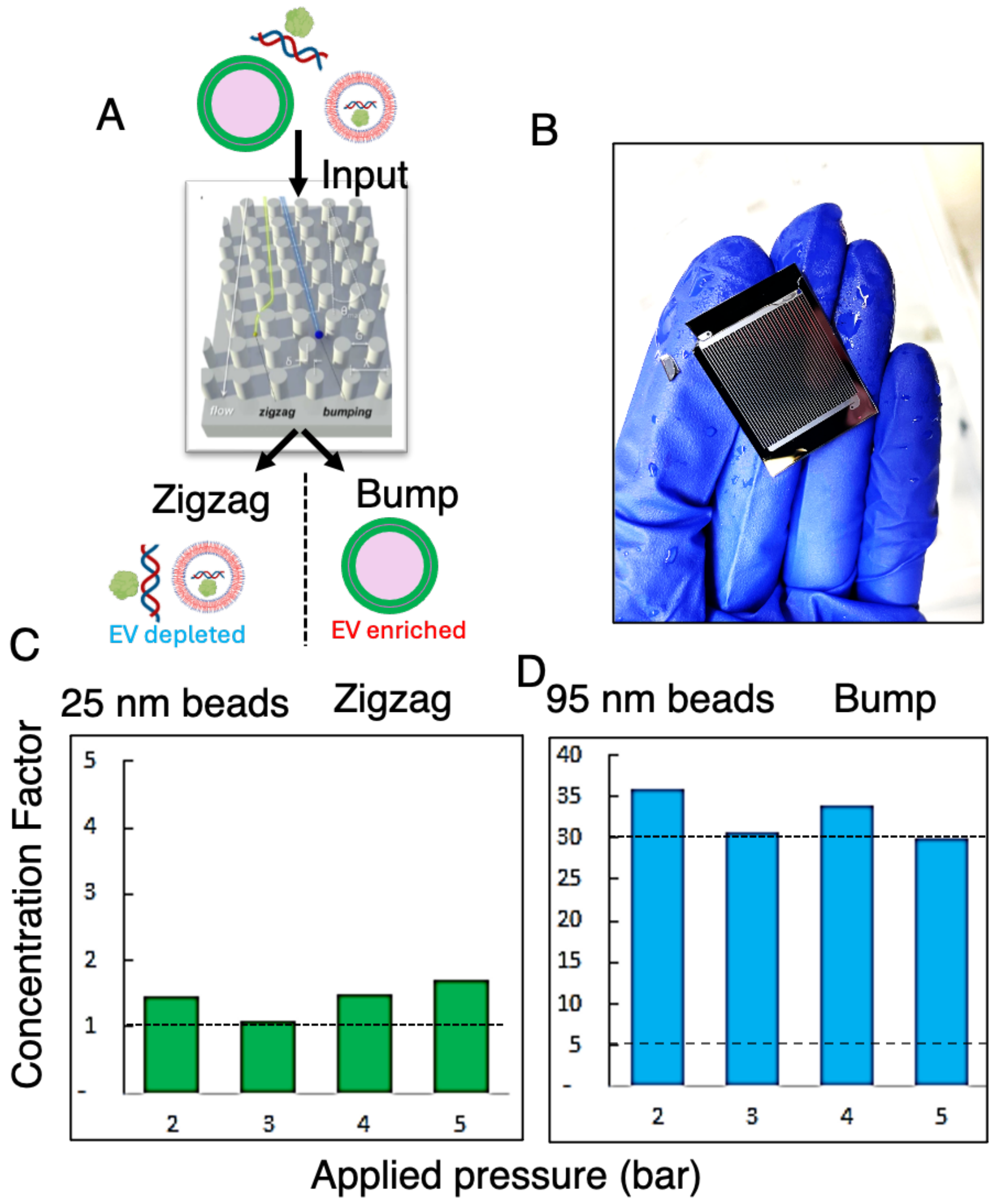

Supplementary Figure 3. A) Overview of nanoDLD chip technology. “Bump” fraction sorts ~100nm particles, while “zigzag” fraction contains particles below ~50nm. B) A picture of nanoDLD chip. C) 25nm beads do not concentrate with nanoDLD. D) 95nm beads concentrate ~35 folds with nanoDLD chip technology.

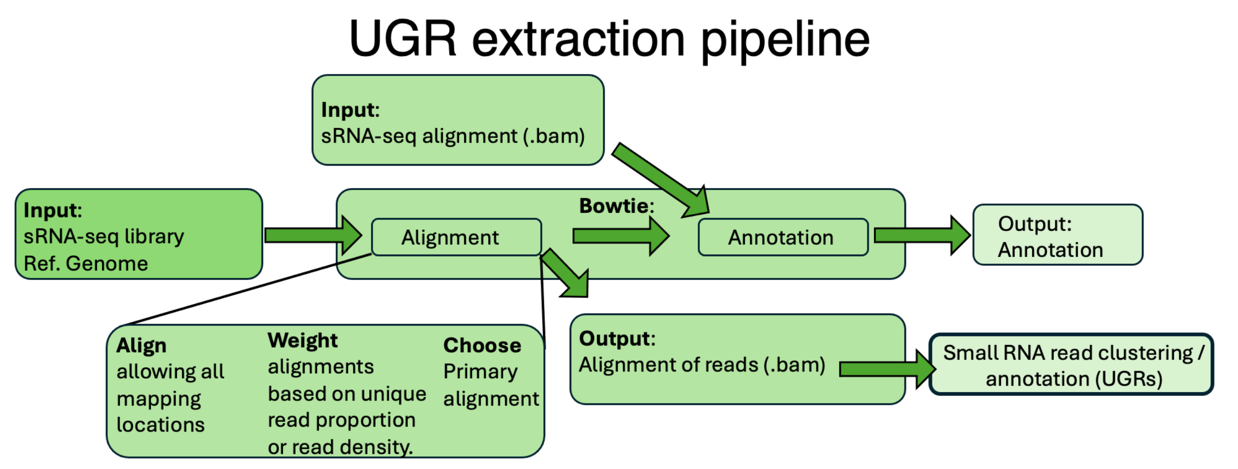

Supplementary Figure 4. UGR identification pipeline.

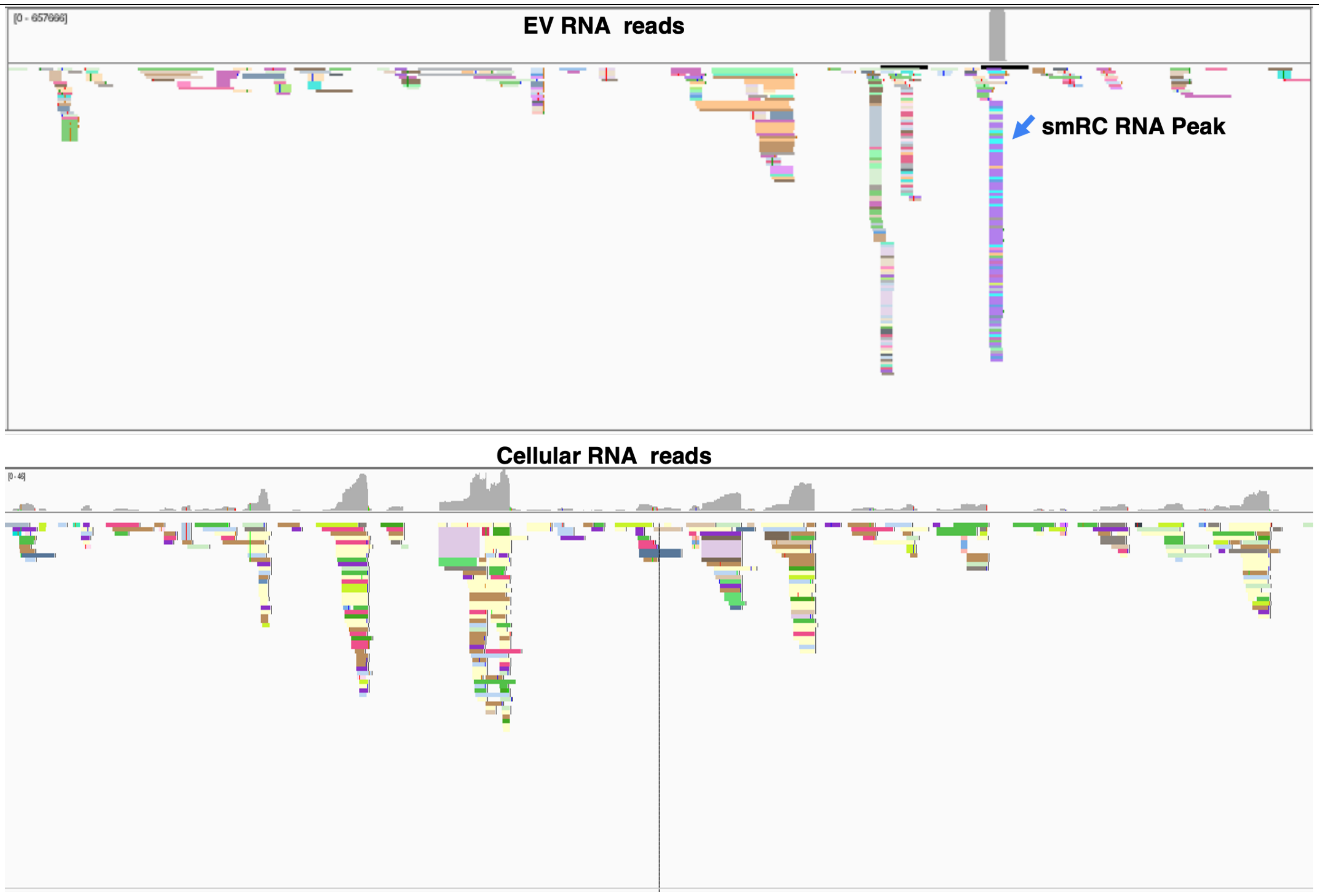

Supplementary Figure 5. Visually investigation of the expression patterns from EV-RNA using Integrative Genome Viewer. EVs display small, sharp, clustered peaks of UGRs (**top panel**), while cells (**lower panel**) display high complexity peaks.

EV-UGR Loci by genomic location				
chr1:156766289-156766835	chr1:28578381-28579056	chr2:148881489-148881928	chr4:72666068-72666179	chr1:53280010-53280210
chr1:22550061-22550183	chr20:2656014-2657249	chr5:136080275-136080898	chr5:181243140-181244018	chr17:38748436-38749040
chr1:2327393-2328202	chr6:156658304-156658527	chr5:160485261-160485451	chr5:697290-697520	chr4:139557440-139557595
chr1:28578381-28579056	chr12:56644732-56646079	chr7:130876600-130876936	chr5:84303189-84303295	chr8:21329709-21329879
chr10:130334670-130334867	chr19:12703120-12703797	chr9:109045847-109046381	chr6:117278658-117278742	chr10:102436160-102436637
chr10:92216302-92216854	chr22:24151093-24151286	chr9:136670119-136671340	chr6:156658304-156658527	chr11:5225396-5225764
chr11:17719432-17720155	chr10:92216302-92216854	chr9:19376090-19377155	chr6:168056970-168057161	chr11:5226898-5227218
chr12:113473778-113473905	chr11:2963676-2963950	chrX:119786266-119786734	chr6:169216934-169217833	chr11:62854427-62855133
chr12:113500430-113500600	chr12:6537548-6538412	chrX:134546004-134546835	chr8:27858124-27858295	chr15:62823860-62824445
chr12:56644732-56646079	chr3:44913579-44914729	chrX:45746066-45746347	chr9:76188316-76188813	chr16:67202182-67202743
chr13:113588776-113589260	chr1:71067600-71067851	chrX:45746661-45747253	chrX:105462325-105462428	chr17:30116645-30117250
chr15:56113733-56114017	chr21:16539681-16539971	chrX:50003142-50003661	chrX:20957905-20958105	chr17:59137601-59138192
chr15:71218226-71218808	chr11:62852588-62854020	chrX:50013156-50013363	chrY:8832787-8832949	chr17:59151007-59151290
chr17:38748436-38749040	chr6:132816687-132817644	chrX:53556121-53556434	chr3:133399970-133400107	chr19:50802219-50802443
chr17:83192866-83193087	chrUn_KI270435v1:92490-92851	chrX:66018748-66019056	chr1:22550061-22550183	chr2:148881489-148881928
chr19:10109664-10110637	chrX:97479181-97479347	chr10:78040543-78040837	chr5:697290-697520	chr2:222918489-222918711
chr19:12703120-12703797	chr1:182751478-182751666	chr11:75399374-75399654	chr19:10109664-10110637	chr3:150541899-150544575
chr19:17180894-17181182	chr1:44778285-44779133	chr11:75405518-75406080	chr5:181243140-181244018	chrX:39509304-39509458
chr19:43596304-43596738	chr12:6962913-6964451	chr12:14774336-14775221	chr4:52712239-52713384	chr7:25949674-25950349
chr2:133147702-133147977	chr16:15642653-15643468	chr20:13823914-13824080	chr6:71403008-71403875	chr8:80036392-80038294
chr2:154041471-154041755	chr17:38852434-38854107	chr20:34534551-34534809	chr6:85677267-85677703	chrX:103586603-103587737
chr2:82587284-82587617	chr17:81183402-81183648	chr3:186784571-186785102	chr8:2974830-2974938	chrX:1385195-1386804
chr20:2656014-2657249	chr20:62008084-62008363	chr4:174490673-174492199	chr8:4134604-4134624	chrX:139923968-139924295
chr21:44433042-44433632	chr3:49019865-49022766	chr7:22812949-22813211	chr8:53744420-53744738	chrX:48577434-48578406
chr22:24151093-24151286	chr3:133399970-133400107	chr3:137891838-137892152	chrX:6813544-6813581	chr13:73260643-73260801
chr3:176727715-176727873	chr3:33430245-33430490	chr3:44913579-44914729	chr4:126431574-126431688	

Supplementary Table. 1. List of identified UGRs loci by their genomic location

Alignment to non-human species and microbiome.

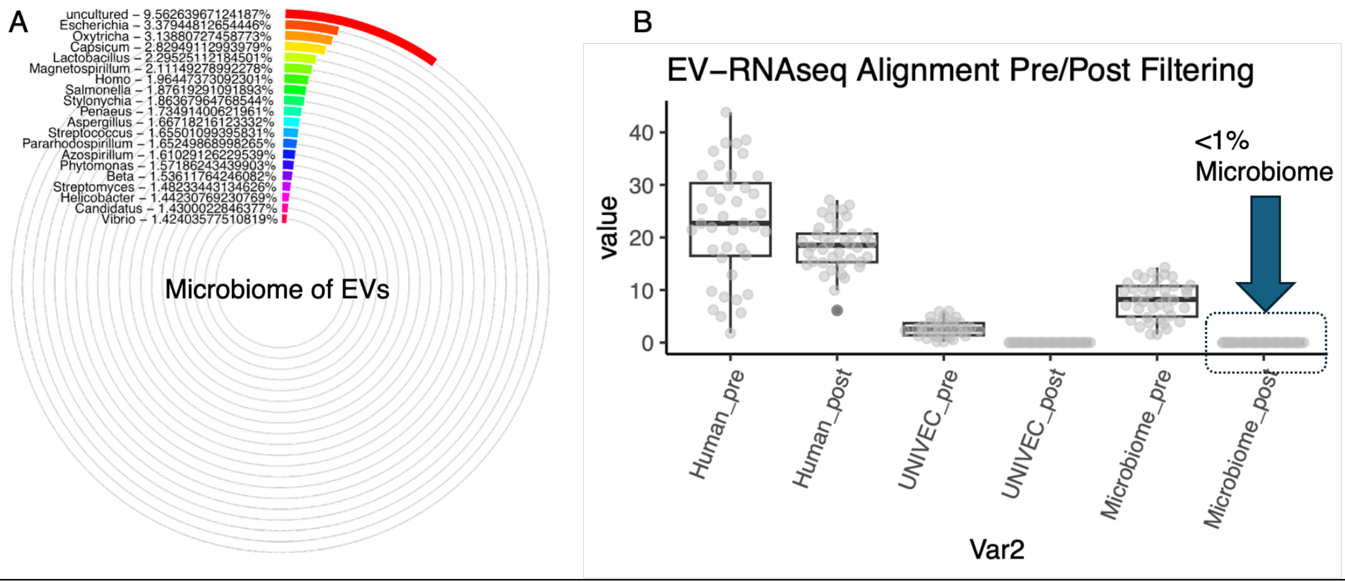

Supplementary Figure 6. Alignment of EV-RNA to non-human species and microbiome. A) Microbiome contribution in EVs transcriptome before filtering. B) Comparison of EV-RNAseq pre- and post-filtering. Arrow shows the EV samples post microbiome-filtering, which is used for EV-UGR detection. Human pre/post: pre- and post-filtering human genome, UNIVEC_pre/post: UniVec is a database that identify segments within nucleic acid sequences which may be of vector origin. Microbiome_pre/post: NIH microbiome database is use for filtering. Post filtering only <1% of the EV transcriptome aligns with microbiome.
