# Supplementary figures and images for: Extracellular vesicles carry transcriptional ‘dark matter’ revealing tissue‐specific information
Source: J Extracell Vesicles. 2024 Aug 15;13(8):e12481. doi: 10.1002/jev2.12481 (PMC11327273; doi:10.1002/jev2.12481)

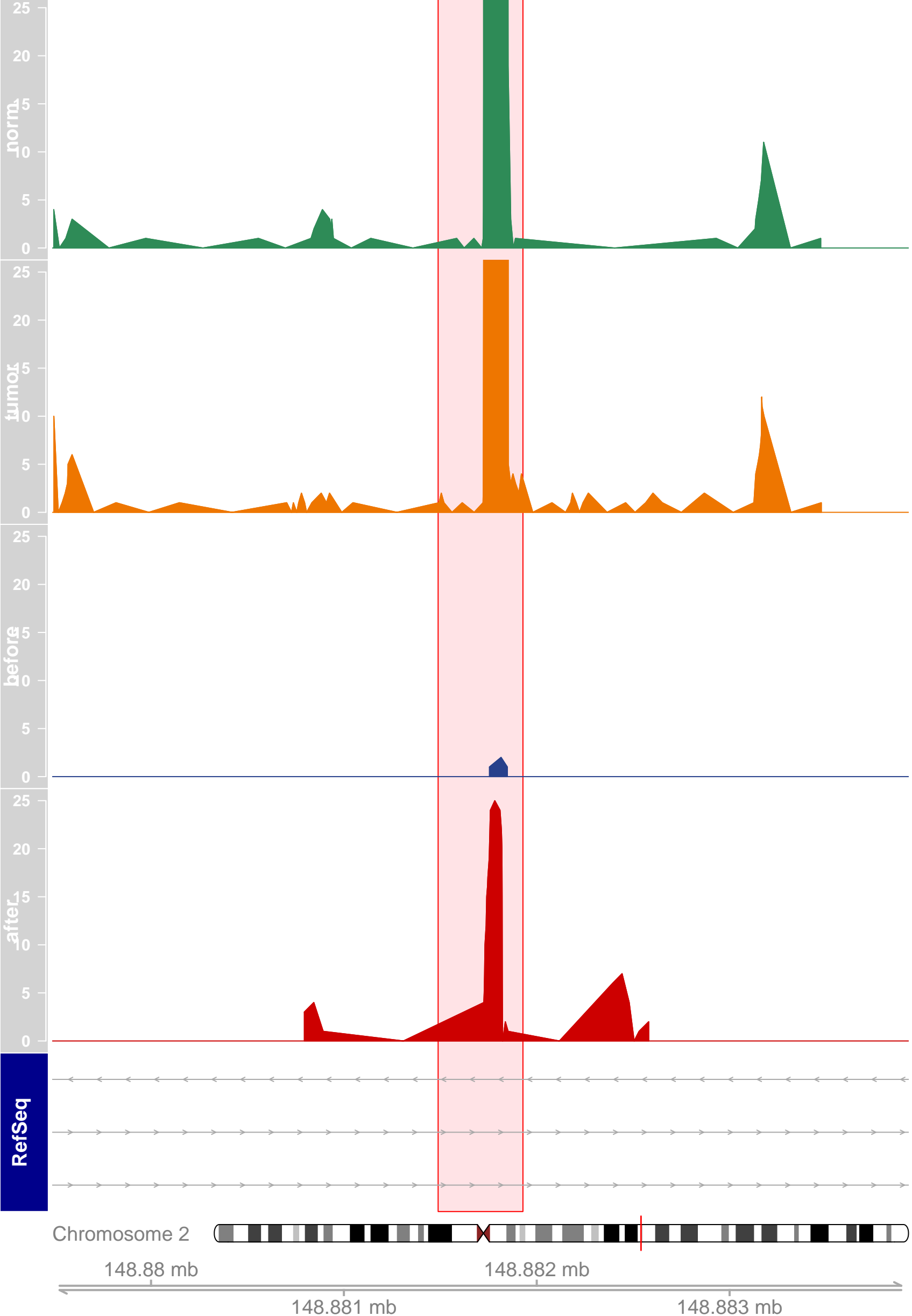

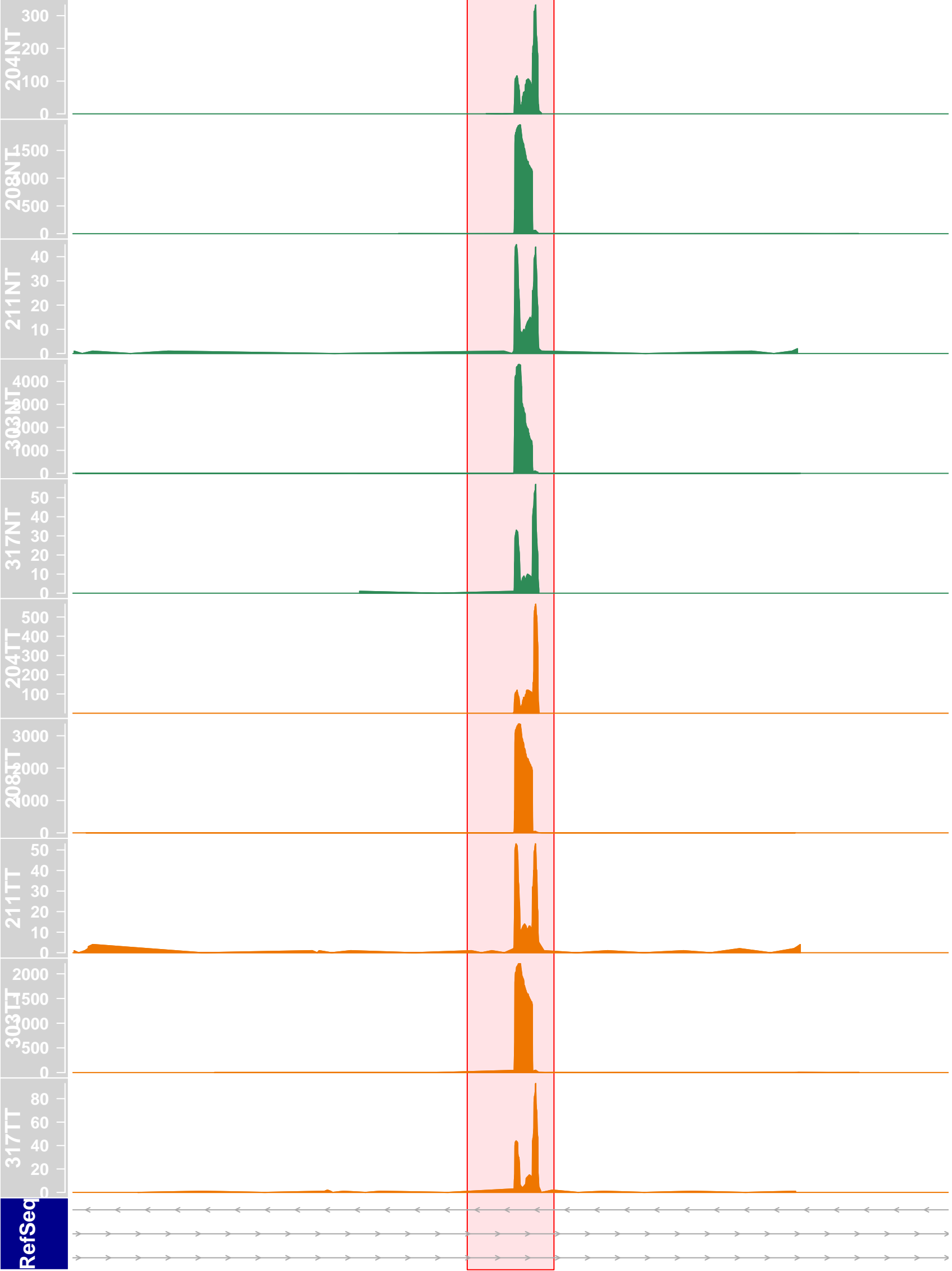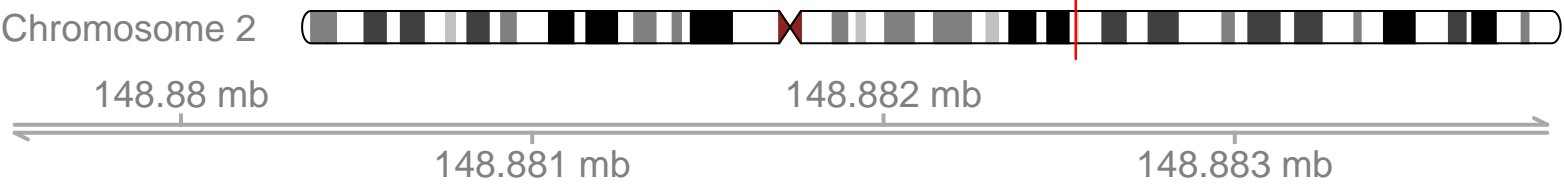

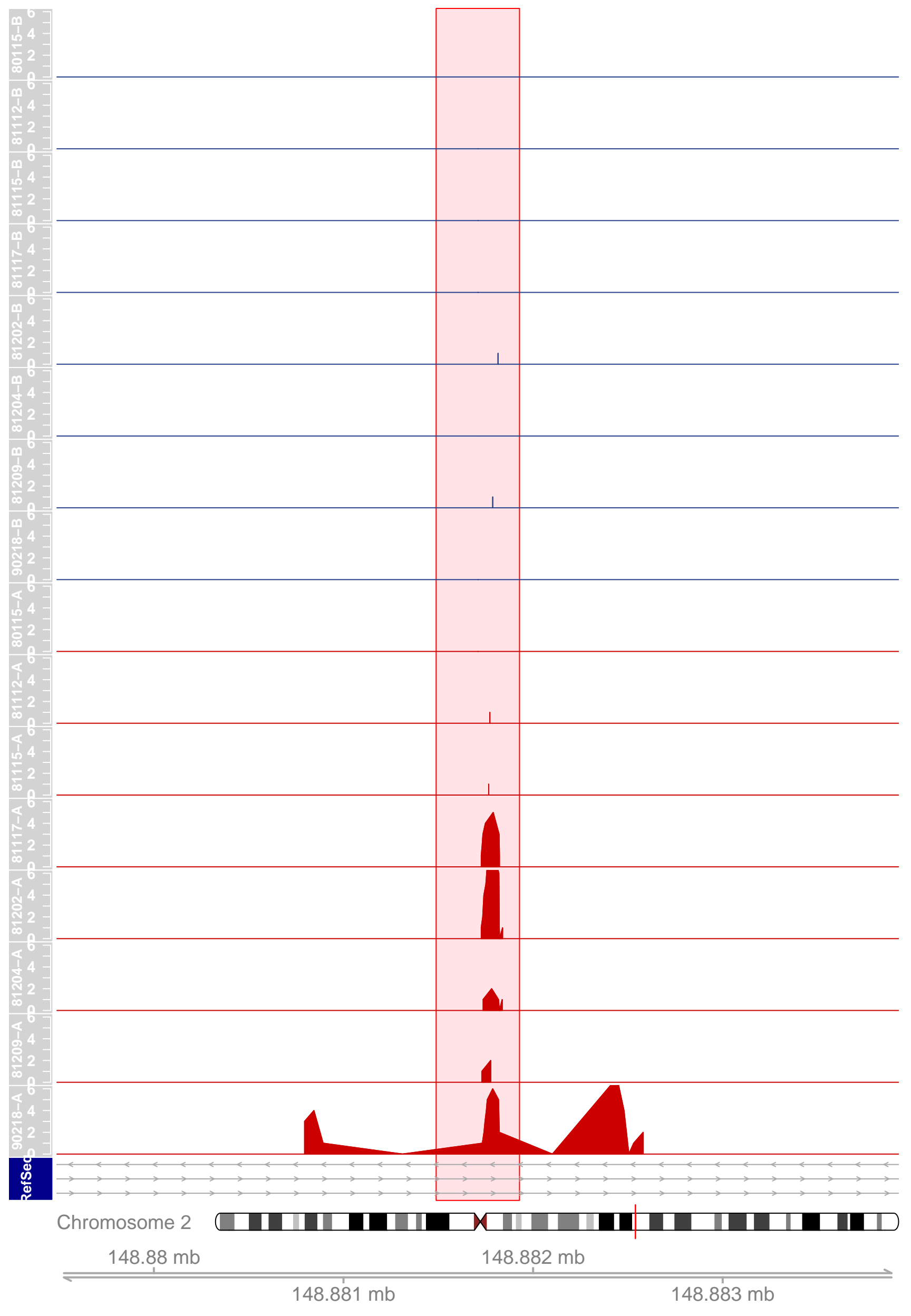

Supplement: Supplementary file 3 — Supporting Information [file JEV2-13-e12481-s003.pdf]

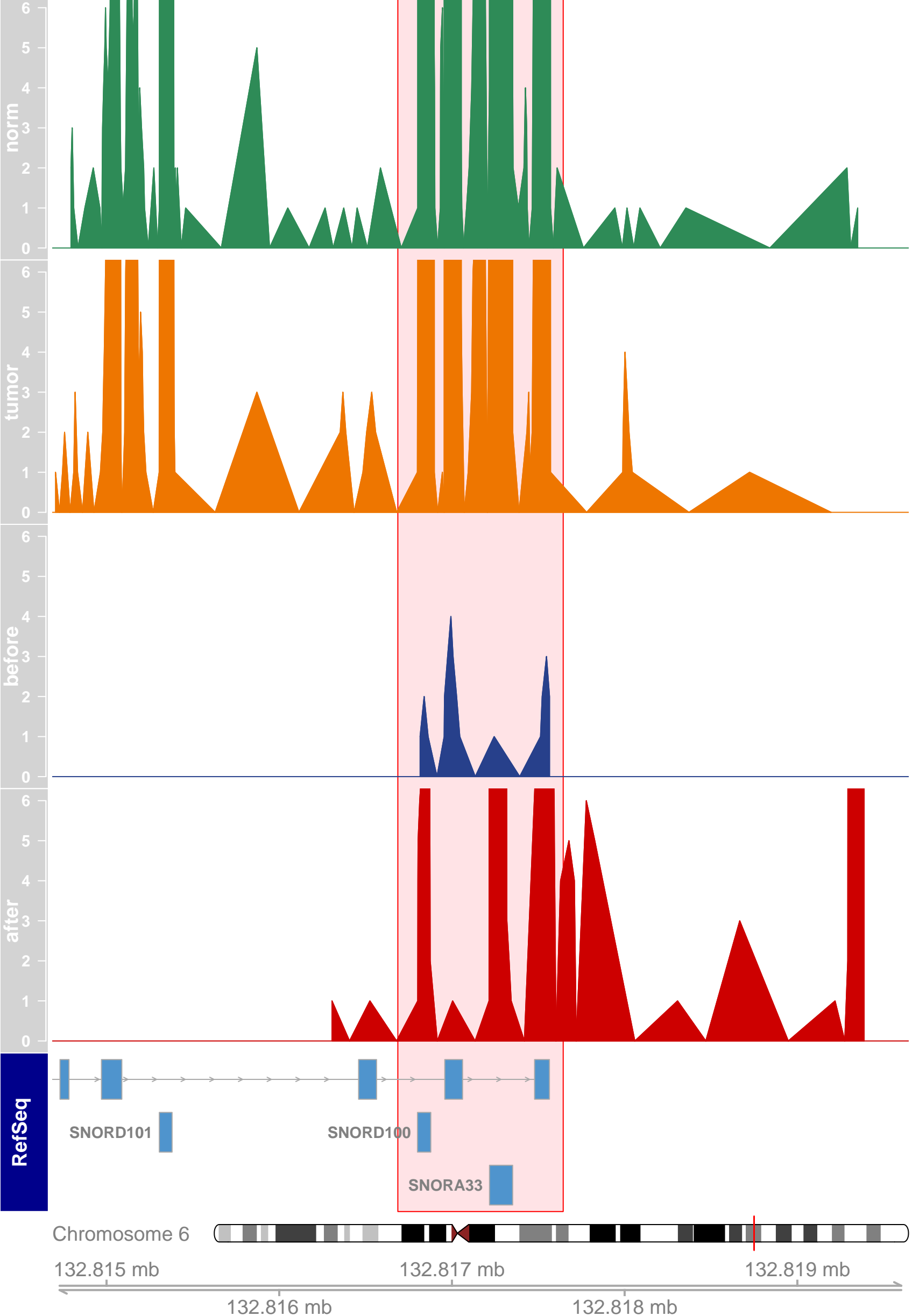

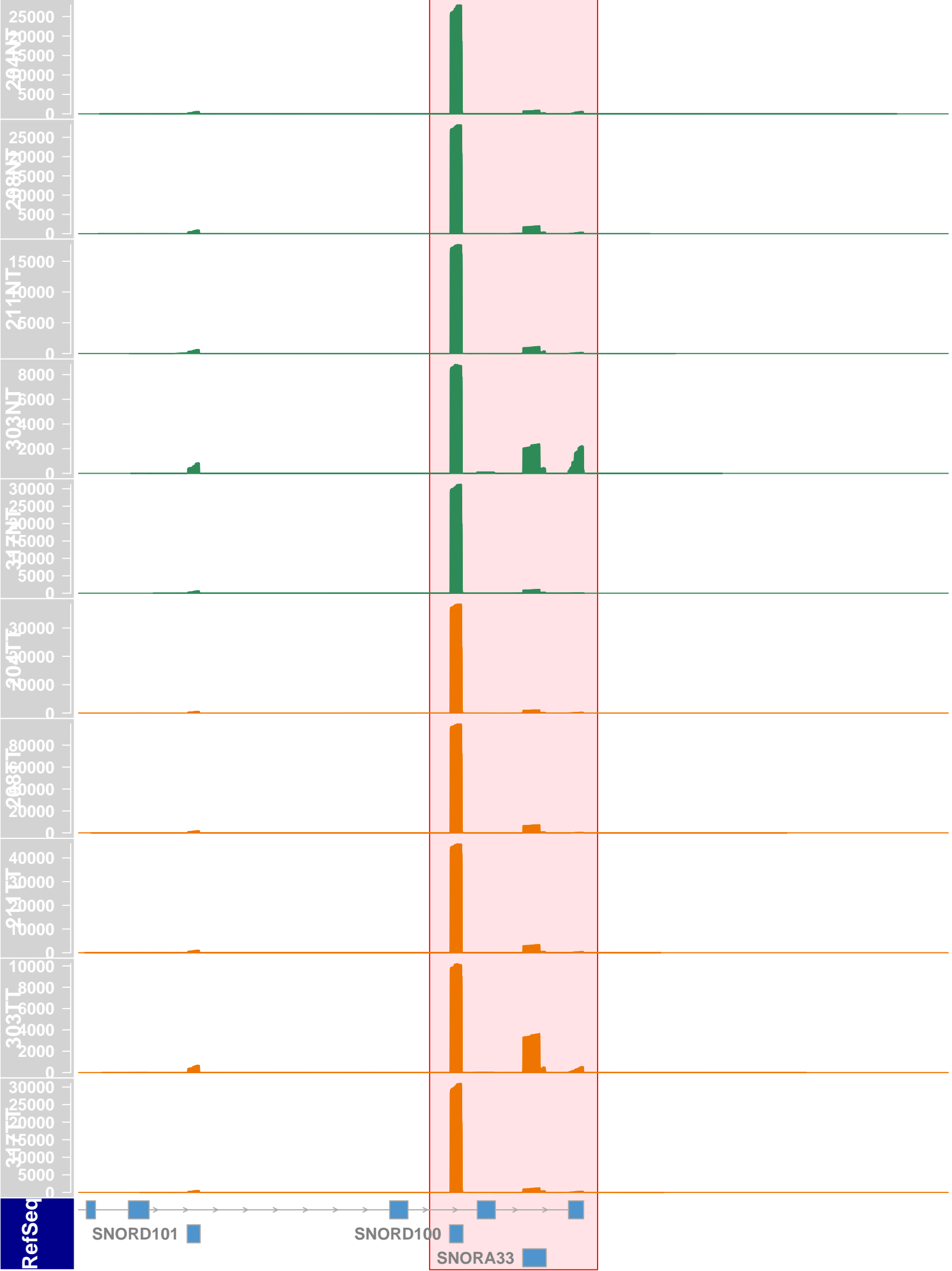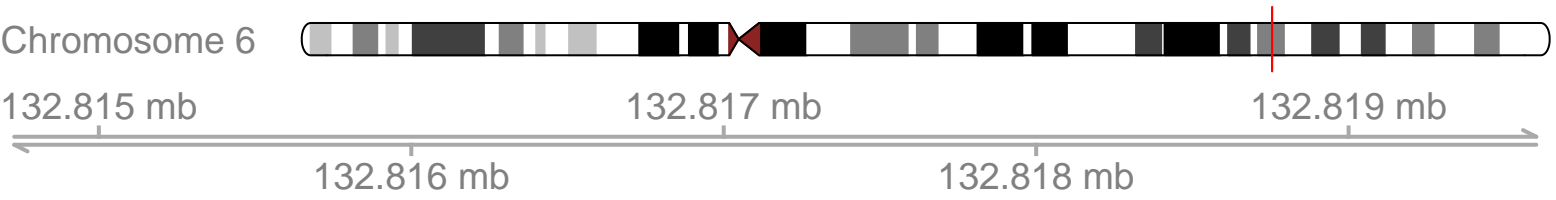

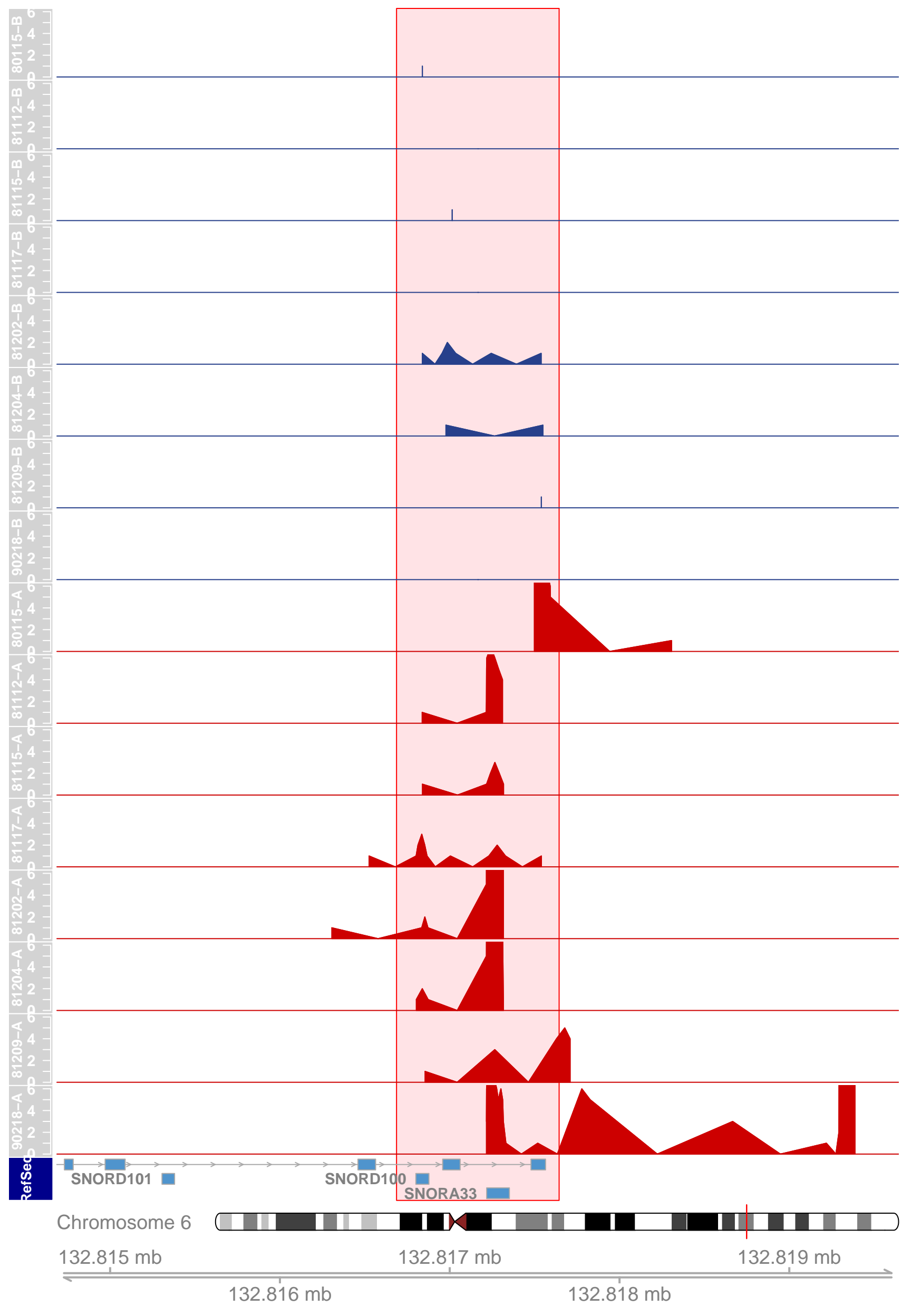

Supplement: Supplementary file 4 — Supporting Information [file JEV2-13-e12481-s005.pdf]

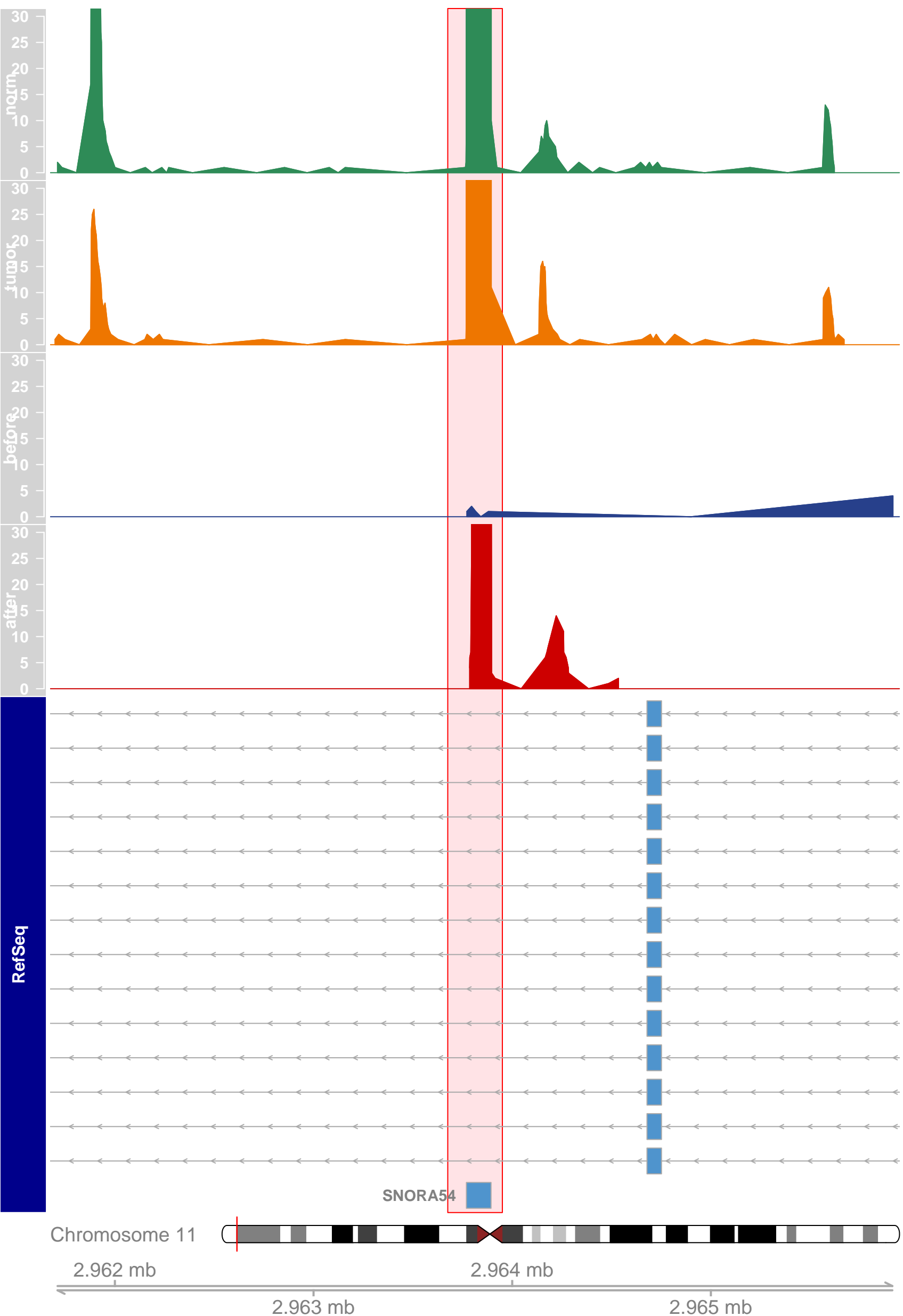

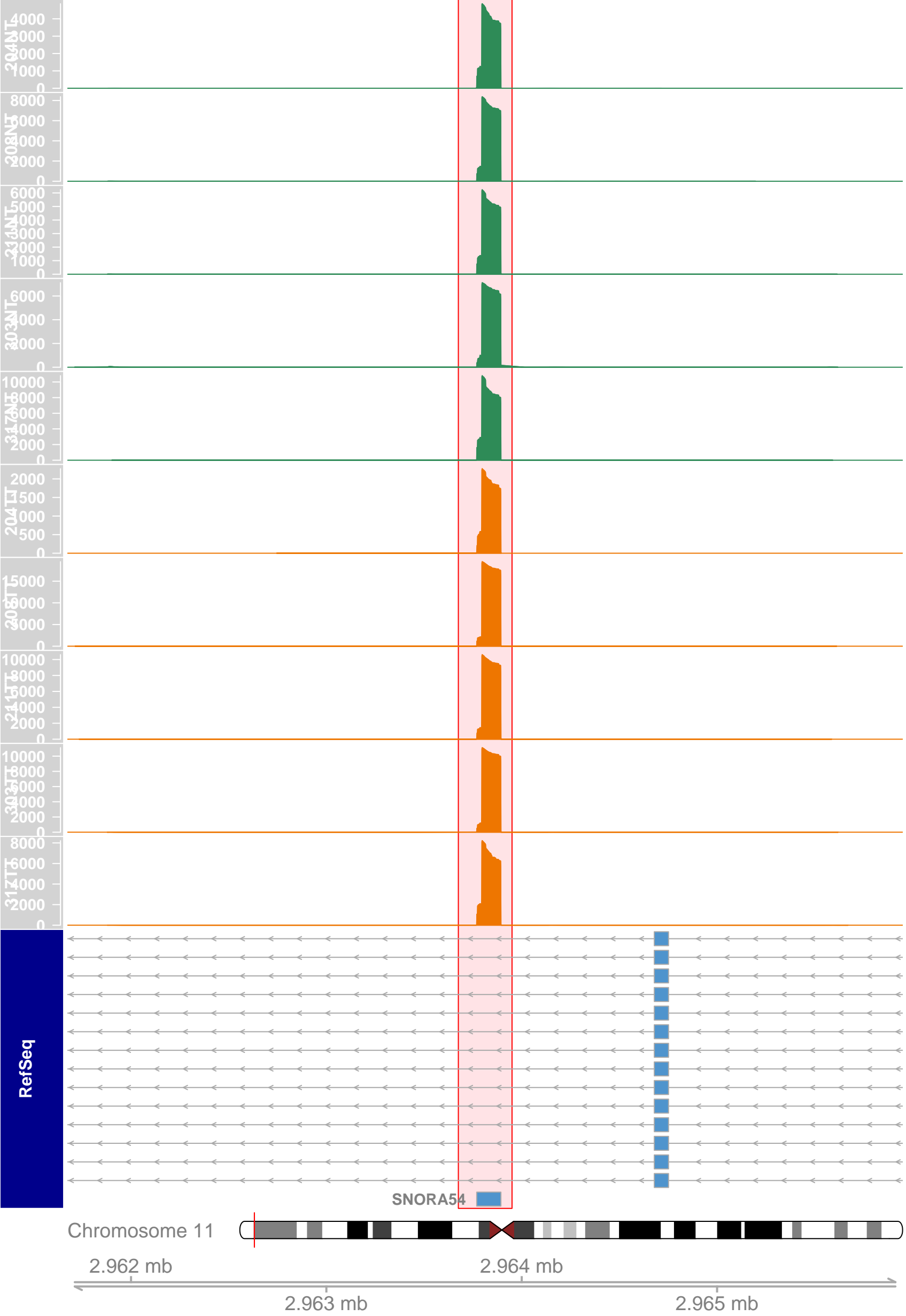

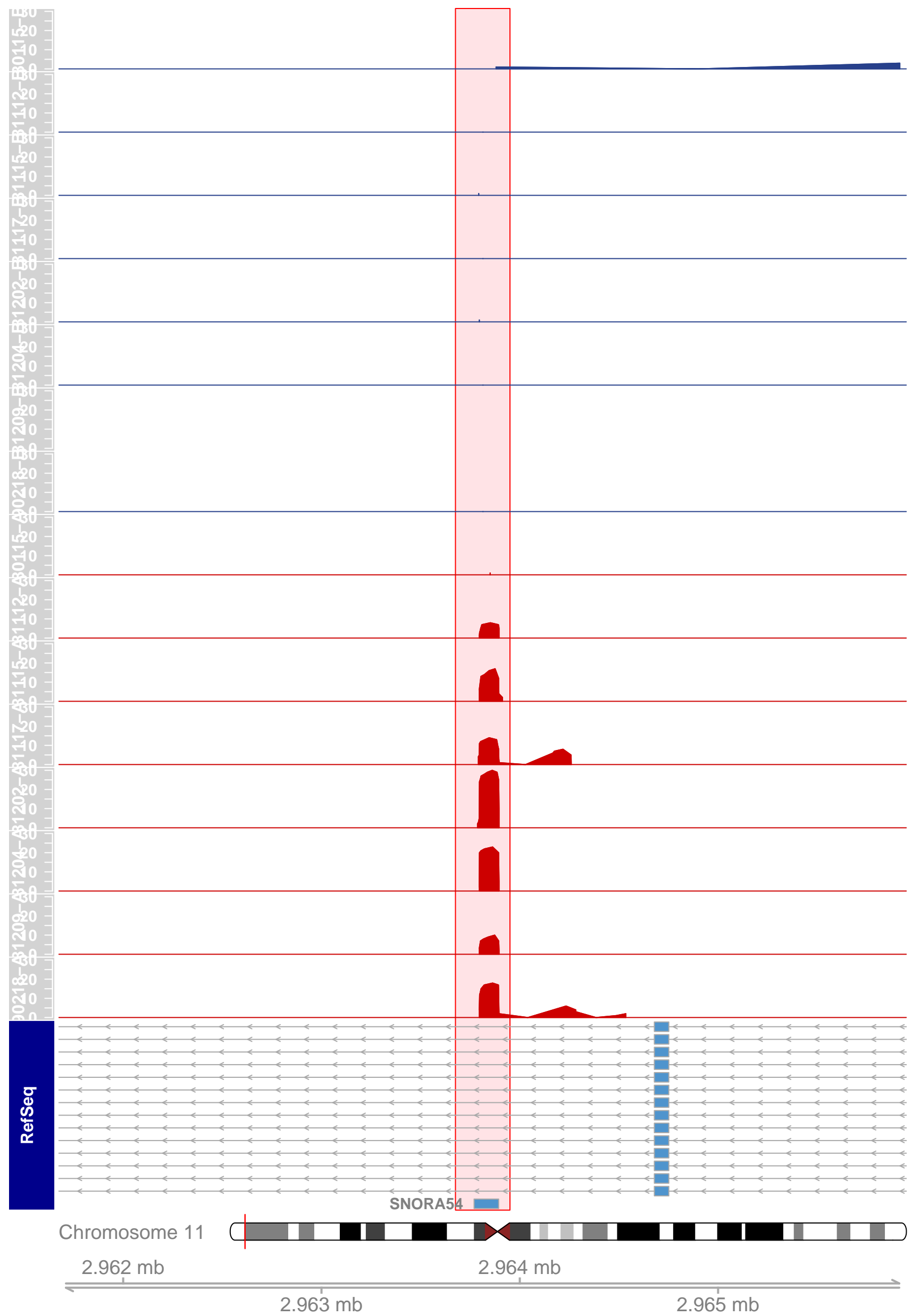

Supplement: Supplementary file 5 — Supporting Information [file JEV2-13-e12481-s001.pdf]
